# X-ray Synchrotron Microtomography of a silicified Jurassic Cheirolepidiaceae (Conifer) cone: histology and morphology of *Pararaucaria collinsonae* sp. nov.

**DOI:** 10.7717/peerj.624

**Published:** 2014-10-23

**Authors:** David C. Steart, Alan R.T. Spencer, Russell J. Garwood, Jason Hilton, Martin C. Munt, John Needham, Paul Kenrick

**Affiliations:** 1 Department of Earth Sciences, Invertebrates and Plants Division, Natural History Museum, London, United Kingdom; 2 Department of Agricultural Sciences, LaTrobe University, Melbourne, Victoria, Australia; 3 Department of Earth Sciences and Engineering, Imperial College London, London, United Kingdom; 4 School of Earth, Atmospheric and Environmental Sciences and The Manchester X-ray Imaging Facility, School of Materials, The University of Manchester, Manchester, United Kingdom; 5 School of Geography, Earth and Environmental Sciences, University of Birmingham, Edgbaston, Birmingham, United Kingdom; 6 Tisbury, Salisbury, Wiltshire, United Kingdom

**Keywords:** *Pararaucaria*, Diamond Light Source, Purbeck, Fossil forest, Tithonian

## Abstract

We document a new species of ovulate cone (*Pararaucaria collinsonae*) on the basis of silicified fossils from the Late Jurassic Purbeck Limestone Group of southern England (Tithonian Stage: ca. 145 million years). Our description principally relies on the anatomy of the ovuliferous scales, revealed through X-ray synchrotron microtomography (SRXMT) performed at the Diamond Light Source (UK). This study represents the first application of SRXMT to macro-scale silicified plant fossils, and demonstrates the significant advantages of this approach, which can resolve cellular structure over lab-based X-ray computed microtomography (XMT). The method enabled us to characterize tissues and precisely demarcate their boundaries, elucidating organ shape, and thus allowing an accurate assessment of affinities. The cones are broadly spherical (ca. 1.3 cm diameter), and are structured around a central axis with helically arranged bract/scale complexes, each of which bares a single ovule. A three-lobed ovuliferous scale and ovules enclosed within pocket-forming tissue, demonstrate an affinity with Cheirolepidiaceae. Details of vascular sclerenchyma bundles, integument structure, and the number and attachment of the ovules indicate greatest similarity to *P. patagonica* and *P. carrii.* This fossil develops our understanding of the dominant tree element of the Purbeck Fossil Forest, providing the first evidence for ovulate cheirolepidiaceous cones in Europe. Alongside recent discoveries in North America, this significantly extends the known palaeogeographic range of *Pararaucaria*, supporting a mid-palaeolatitudinal distribution in both Gondwana and Laurasia during the Late Jurassic. Palaeoclimatic interpretations derived from contemporaneous floras, climate sensitive sediments, and general circulation climate models indicate that *Pararaucaria* was a constituent of low diversity floras in semi-arid Mediterranean-type environments.

## Introduction

The Cheirolepidiaceae were a distinctive and diverse group of conifers. They are well-documented through an extensive Mesozoic fossil record that includes foliage, wood, cones, and most notably pollen of the *Classopollis* type (Alvin, 1982; Watson, 1988; Clement-Westerhof & Van Konijnenburg-Van Cittert, 1991; Taylor, Taylor & Krings, 2009). Many species were large trees, but the family is thought to encompass a broad range of growth forms, including woody shrubs and possibly herbs. Due to the fragmentation of parts, however, direct evidence of habit is rare (Axsmith & Jacobs, 2005). In contrast to modern conifers, Cheirolepidiaceae were widespread in coastal environments at low to mid palaeolatitudes, especially during the Cretaceous Period (Lupia, Lidgard & Crane, 1999). Evidence from sediments and cuticle morphology, most notably the sunken papillate stomata (Watson, 1988), indicate that many were adapted to xeric habitats and that they grew in brackish coastal mires (Batten, 1974; Watson, 1988) and on the margins of freshwater rivers and lakes (Gomez et al., 2002). In fact, their ecological dominance in these environments was a unique and distinctive feature of late Mesozoic floras (Watson, 1988; Coiffard et al., 2006). Many aspects of Cheirolepidiaceae morphology and habit remain poorly understood. In particular the structure of the ovulate cones has proven difficult to discern due to a predominance of compression fossils, sometimes with internal cuticular membranes preserved (Clement-Westerhof & Van Konijnenburg-Van Cittert, 1991). Permineralized cones have only recently been recognised, providing important new insights into their internal tissue systems (Escapa et al., 2012; Escapa et al., 2013; Stockey & Rothwell, 2013).

The ovuliferous cones of the Cheirolepidiaceae are rare; where documented they are assigned to the following principal genera (*Alvinia* Kvaček, *Kachaikestrobus* Del Fueyo et al., *Pararaucaria* Weiland, *Pseudohirmeriella* Arndt, *Pseudofrenelopsis* Nathorst and *Hirmeriella* Hörhammer). Of these the oldest is *Hirmeriella* from Upper Triassic to Lower Jurassic (ca. Rhaetian/Hettangian) deposits of Germany and Wales (Jung, 1967, Jung, 1968; Clement-Westerhof & Van Konijnenburg-Van Cittert, 1991). The genus *Pararaucaria* was erected by Wieland (1929) and Wieland (1935) to accommodate permineralized ovuliferous cones from the Middle Jurassic Cerro Cuadrado Petrified Forest (Patagonia, Argentina). *Pararaucaria* has a chequered taxonomic history, with attributions to various families, including Araucariaceae (Wieland, 1929; Wieland, 1935; Calder, 1953), Cheirolepidiaceae (Wieland, 1935; Archangelsky, 1968), Pinaceae (Stockey, 1977; Smith & Stockey, 2001; Smith & Stockey, 2002) and Voltziaceae (Miller, 1999). Previous interpretations of the type species (*Pararaucaria patagonica*) differentiated it from Cheirolepidiaceae on the apparent absence of an adaxial flap of tissue enclosing the ovules, the apparent presence of a wing to the ovule (Stockey, 1977), and the common occurrence of a single ovule per scale, as opposed to two, which is more typical in the family (Jung, 1968; Clement-Westerhof & Van Konijnenburg-Van Cittert, 1991; Del Fueyo et al., 2008). A recent critical reinterpretation based on well-preserved petrified material now affirms a relationship with Cheirolepidiaceae, by clarifying crucial aspects of cone anatomy (Escapa et al., 2012). Additional cone species *P. delfueyoi* from the Late Jurassic Cañadón Calcáreo Formation (Argentina) (Escapa et al., 2013), *P. carrii*, from the Middle Jurassic of Oregon (USA) (Stockey & Rothwell, 2013), and a cone morphotype assigned to *Pararaucaria* from the Late Jurassic Morrison Formation (USA) (Gee et al., 2014) have recently added further information about the group. A layer of adaxial spongy tissue forming an open seed-enclosing pocket (pocket forming tissue), which is unique to the family, is now recognised as a key feature. Other defining characteristics—as previously established or emended by Escapa et al. (2012)—include the presence of lobes on the ovuliferous scale, combined with a subtending bract that is free for much of its length (Escapa et al., 2012; Escapa et al., 2013). These authors also emphasised the presence of prominent sclerenchyma strands, triangular in cross section at the basal region of the ovuliferous scale, that flank the scale trace (Escapa et al., 2012).The ovuliferous scale complexes bear one broad wingless inverted ovule each, with two known exceptions. The first occurs in a single *P. delfueyoi* cone, where ovuliferous scale complexes containing two ovules were found on only one side of the cone axis (Escapa et al., 2012; GW Rothwell, pers. comm., 2014). The second exception involves an unnamed cone with two ovules per ovuliferous scale complex that was recovered from the Morrison Formation, which Gee et al. (2014) have preliminarily assigned to the *Pararaucaria*. Accurate interpretation of the tissue and organ structure within petrified cones is therefore essential to determining their systematic affinity.

Here, we investigate the internal structure of a new permineralized cone species using Synchrotron X-ray Microtomography (SRXMT) at the Diamond Light Source (UK). SRXMT is one of a suite of X-ray tomographic techniques that are now widely employed in palaeontology to produce high resolution three-dimensional virtual histological models of the internal structures and anatomy of fossils (Collinson et al., 2012; Sutton, Rahman & Garwood, 2014). In the context of fossil plants this approach is favoured because it is non-invasive. Nevertheless, the resulting image stacks can be processed to reconstruct volumes showing internal structures (including distinct organs and tissues) in a manner comparable to traditional methods involving thin sections or peels (e.g., DeVore et al., 2006; Sanchez et al., 2012; Gee, 2013). SRXMT is especially suited to studies of tissue systems within small objects because it can resolve features at cellular to subcellular levels (e.g., Friis et al., 2007; Scott et al., 2009; Smith et al., 2009; Strullu-Derrien et al., 2014), which is a result of the technique’s significantly higher resolution than the more versatile and widely available X-ray Microtomography (XMT; Collinson et al., 2012). The requirement for cellular level resolution, to facilitate the detailed structural, anatomical and functional interpretation of internal tissues and organs, dictated our choice of method. We also wished to avoid destructive sectioning of a unique specimen. Our results extend the application of SRXMT to plant fossils permineralized in silicates. The novel species description is based entirely on this non-invasive and non-destructive method.

## Material & Methods

### Locality and geology

**Figure 1:**
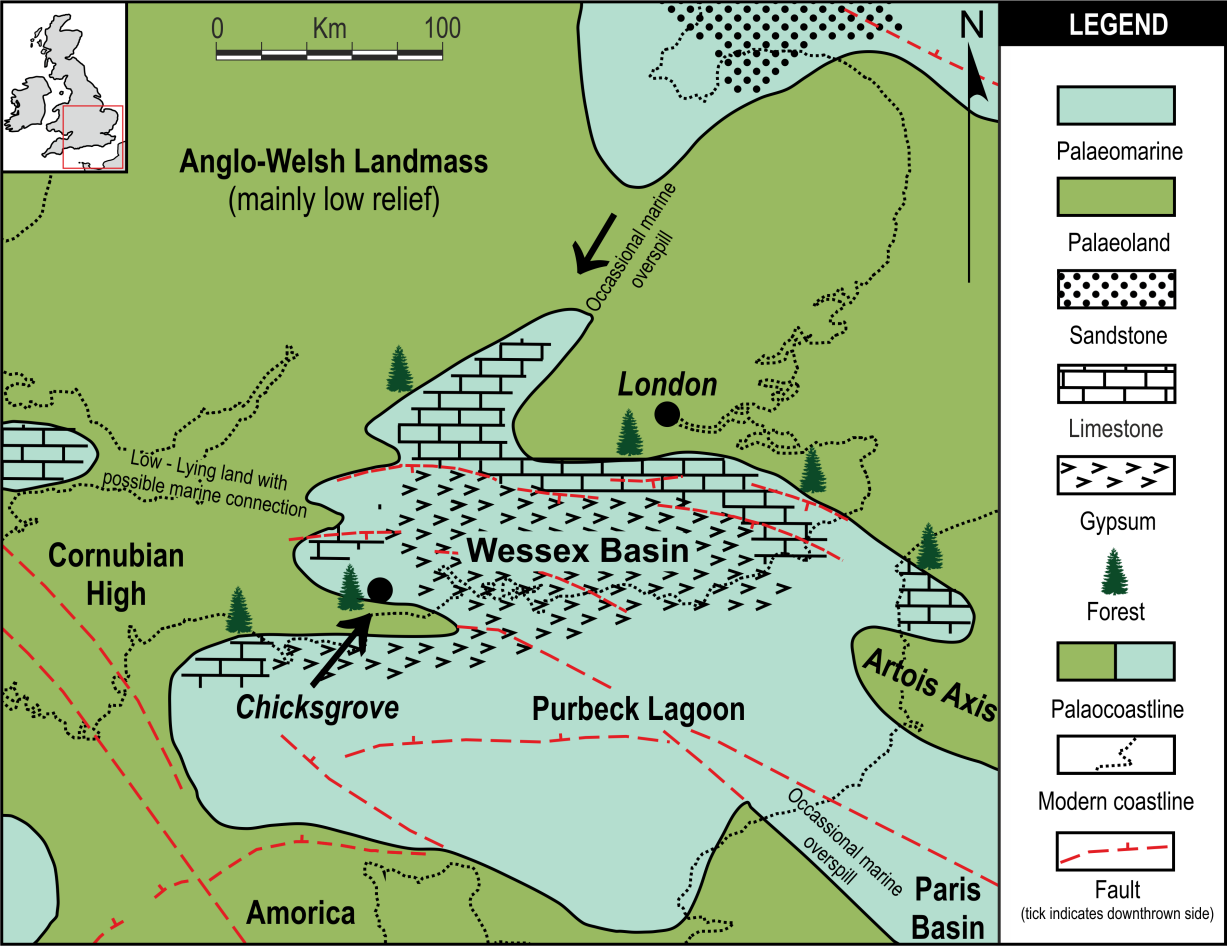
Local palaeogeographic map showing position of Chicksgrove in relation to the Purbeck Lagoon, and the final Jurassic Purbeck regression with evaporites in Southern England. Modified from Bradshaw et al. (1992).

**Figure 2:**
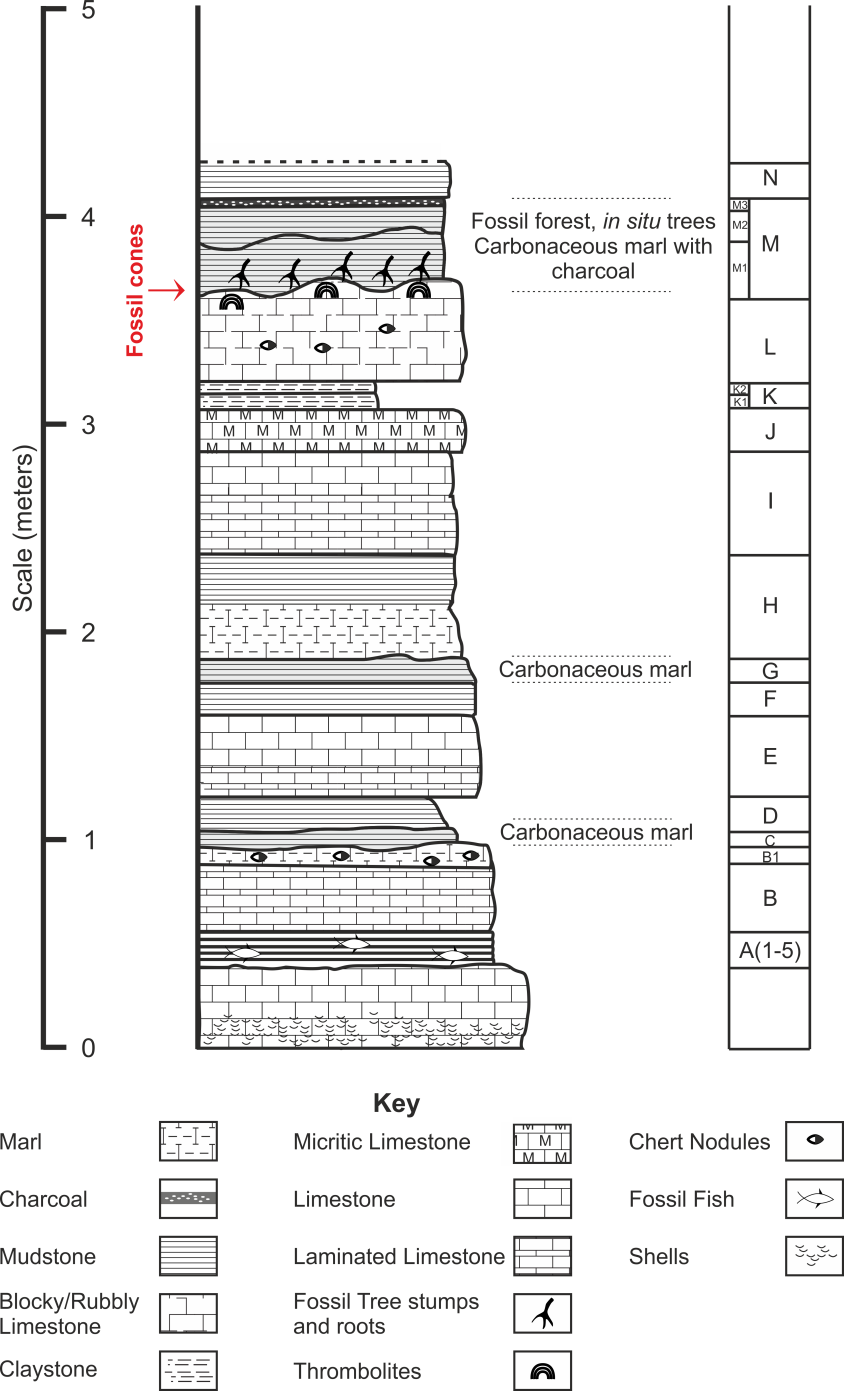
Stratigraphic log from showing position of the Great Dirt Bed within the Purbeck Formation as seen at Chicksgrove Quarry. Bed descriptions: A, Five bands of grey coloured hard laminated marl with fish remains; B, Millimetre scale cream/grey laminated limestone, marly at top with chert nodules; C, Grey/Brown mud (Palaeosol); D, Light grey/brown mud; E, Light cream coloured blocky limestone laminated at the base, chert nodules near top; G, Dark brown mud (Palaeosol); H, Cream to grey coloured centimetre scale laminated limestone; I, Hard creamy-white limestone, laminated at base; J, Hard grey micrite limestone; K, Laminated yellow/brown mud; L, Hard blocky limestone grey at top, chert nodules; M, Grey/brown muds with charcoal layer at top (Palaeosol); N, Creamy yellow/brown mud.

Chicksgrove Quarry is a working building-stone quarry situated in the Vale of Wardour about 2 km east of Tisbury, Wiltshire (UK) (51.07N, −2.05W; Fig. 1). The site exposes a 27 m thick succession of Portland Limestone, which is overlain by basal beds of the Purbeck Limestone Group (Lulworth Formation) (Benton, Cook & Hooker, 2005; Wimbledon, 1976). The rocks are attributed to the latter part of the Tithonian Stage (Jurassic Period), known locally as Portlandian (Wright & Cox, 2001; Benton, Cook & Hooker, 2005; Cope, 2008), which approximates to an age of between 145 and 152 million years. The stratigraphy of the quarry was described in detail and the succession was correlated with other Portlandian sequences in the UK by Wimbledon (1976) and Wimbledon (1980). Excavations in the 1980s yielded a diverse fauna with associated floral elements from a stratigraphic unit called the plant-reptile bed, which is located within the marine Portland Group (= Bed 25 of Wimbledon, 1976). In 2008, commercial stripping of surface sediments in preparation for the opening of a new quarry face exposed an area of the Lulworth Formation containing the *in situ* remains of a petrified forest of upright stumps and fallen trunks of large conifer trees and small Bennettitalean shrubs (Needham, 2011). Our generalized section shows that the exposed forest sits on top of some 4 m of basal Purbeck Limestone (Fig. 2), which is 8 m above the plant-reptile bed of earlier excavations. The base of this section is the base of the Lulworth Formation (i.e., base of Purbeck Limestone = Bed 34 of Wimbledon, 1976), and it is marked by a transition from a distinctive oolitic bioclastic limestone containing numerous shells of the bivalves *Protocardia* and *Camptonectes* into a sequence of mostly thin-bedded pelletoidal limestones and hummocky thrombolytic limestones. Several beds contain conspicuous black chert nodules. The section also contains occasional bands of thin laminated marly clays, and we recognise three distinctive thin carbonaceous marls. The best developed of these is associated with the *in situ* remains of the petrified forest, where it is of variable thickness and draped over a hummocky thrombolytic limestone surface (Fig. 2, band M). This represents a palaeosol, which we tentatively equate with either the Great Dirt Bed or the Lower Dirt Bed of basal Purbeck facies elsewhere in southern England (e.g., Francis, 1983; Francis, 1984). The marl contains charcoal, but unlike the Great Dirt Bed, limestone pebbles were not observed. The specimens documented (NHMUK V 68524 and NHMUK V 68525) are silicified. They were collected from fissures within the thrombolytic limestone by John Needham and are housed in The Natural History Museum, London (UK).

#### Methodology

The internal structures of the cones were first probed using XMT on a Nikon Metrology HMX-ST 225 scanner at the Natural History Museum London. The scan was performed with a tungsten reflection target at 180 µA and 130 kV, 0.25 s exposure time for 6284 projections. No filter was used. The 4 MP (2,000 × 2,000) Perkins Elmer detector panel provided a voxel size of 8.8 µm for specimen NHMUK V 68524 and 6.5 µm for the compressed cone (specimen NHMUK V 68525). The results from the XMT were sufficient to demonstrate the high quality of the internal preservation and the ovulate nature of the cone, however the compressed cone had poor attenuation contrast and a greatly distorted internal anatomy so was not investigated further. In order to achieve improved resolution and observe crucial cellular detail, additional scans of specimen NHMUK V 68524 were performed at the Diamond Light Source (DLS) synchrotron. The I12—JEEP (Joint Engineering, Environment and Processing)—beamline was selected for its high brilliance and ability to perform high resolution scans on macro-sized objects. Experiment Hutch (EH1) was used with a monochromatic beam set at 70 KeV with a cadmium tungstate crystal scintillator capturing attenuation contrast images on a PCO.4000 camera. The second scan used Module 2, with 0.5 s exposure time for 1,800 projections, providing a voxel size of 5.0 µm and a field of view of 20 × 13.2 mm. The third very high resolution scan used Module 3 through a quadrant of the cone base, with an increased exposure time of 2.5 s for 1,800 projections, generating a voxel size of 1.8 µm with a field of view 7.2 × 4.8 mm.

Due to their higher resolution, the SRXMT datasets acquired at DLS were subsequently selected as the basis for three-dimensional models created with the SPIERS software suite (Sutton et al., 2012). Following the methods described by Spencer, Hilton & Sutton (2013) and Sutton et al. (2012), false colour models illustrating the locations, form and interrelationships between various anatomical features were produced. Raytraced images and videos of the three-dimensional models where composited and rendered using the open source three-dimensional animation package Blender™ (www.blender.org; Garwood & Dunlop, 2014). The images used in figures/plates were edited and processed (cropped, rotated, edge enhanced and equalised) in GIMP 2, ImageJ (Abràmoff, Magalhães & Ram, 2004) and Corel Paint Shop Pro Photo X2™ with figures constructed in Adobe Illustrator CS5™, Corel Creative Suite X5™ and Inkscape 0.48. The tomographic datasets are available upon request from the Natural History Museum (London).

A literature survey was conducted to plot the palaeogeographic distribution of all published pollen- and seed-cones of the Cheirolepidiaceae. Palaeogeographic latitude and longitudes were calculated using PointTracker after determining modern geographical co-ordinates from topographic and geological maps in tandem with Google Earth™. These were plotted using Corel Creative Suite X5™ on to a series of Mesozoic palaeomaps showing individual time slices. The palaeomaps were based on global palaeo-reconstruction maps by Ron Blakey (Blakey, 2008). See Data S1.

## Systematic Description

**Table:** 

*Class*—Spermatopsida
*Order*—Coniferales Florin
*Family*—Cheirolepidiaceae Takhtajan
*Genus*—*Pararaucaria* Wieland emend. Escapa, Rothwell, Stockey et Cúneo 2012
*Species*—*Pararaucaria collinsonae*, Steart, Spencer, Kenrick, Needham, et Hilton sp. nov.

*Specific diagnosis.* Cone broadly spherical (ca. 1.3 cm diameter). Axis with helically arranged bract/scale complexes consisting of a large three lobed ovuliferous scale, subtended by a broad, ‘gull-wing’-shaped bract. Bract and scale separating near base. Scale trace flanked by two connected triangular sclerenchyma bundles within bract and basal region of ovuliferous scale. Adaxial pocket-forming tissue towards apex of ovuliferous scale which overarches and encloses the ovules with an opening at the proximal end of the seed scale complex. The pocket-forming tissue thickens distally, thinning towards scale base, terminating near seed micropyle leaving the pocket opening towards cone axis. The bract/scale traces diverge from the stele at ca. 90°, as two separate bundles forming an inverted ‘U’-shaped scale trace over an ovoid bract trace as they reach the middle zone of the bract cortex. The scale trace forms a vascular pad at the distal end of pocket forming tissue for ovule vascularisation, subsequently branching distally laterally and vertically towards zones of transfusion tissue. Bract trace diverges into multiple vascular bundles accompanied by a band of transfusion tissue distally. One ovule per scale. Ovules rounded, not cordate, 3–4 mm wide and 3–4 mm long and ca. 2 mm thick. Integument with outer thin sarcotesta, sclerotesta of dark cells in general alignment with the ovule axis and an inner unicellular endotesta.

*Type horizon*. Bed ‘M’ (Fig. 2), Chicksgrove Quarry, Purbeck Group, Lulworth Formation. Tithonian Stage, Upper Jurassic.

*Type Locality*. Chicksgrove Quarry, England (N 51.07°, –W 2.05°).

**Figure 3:**
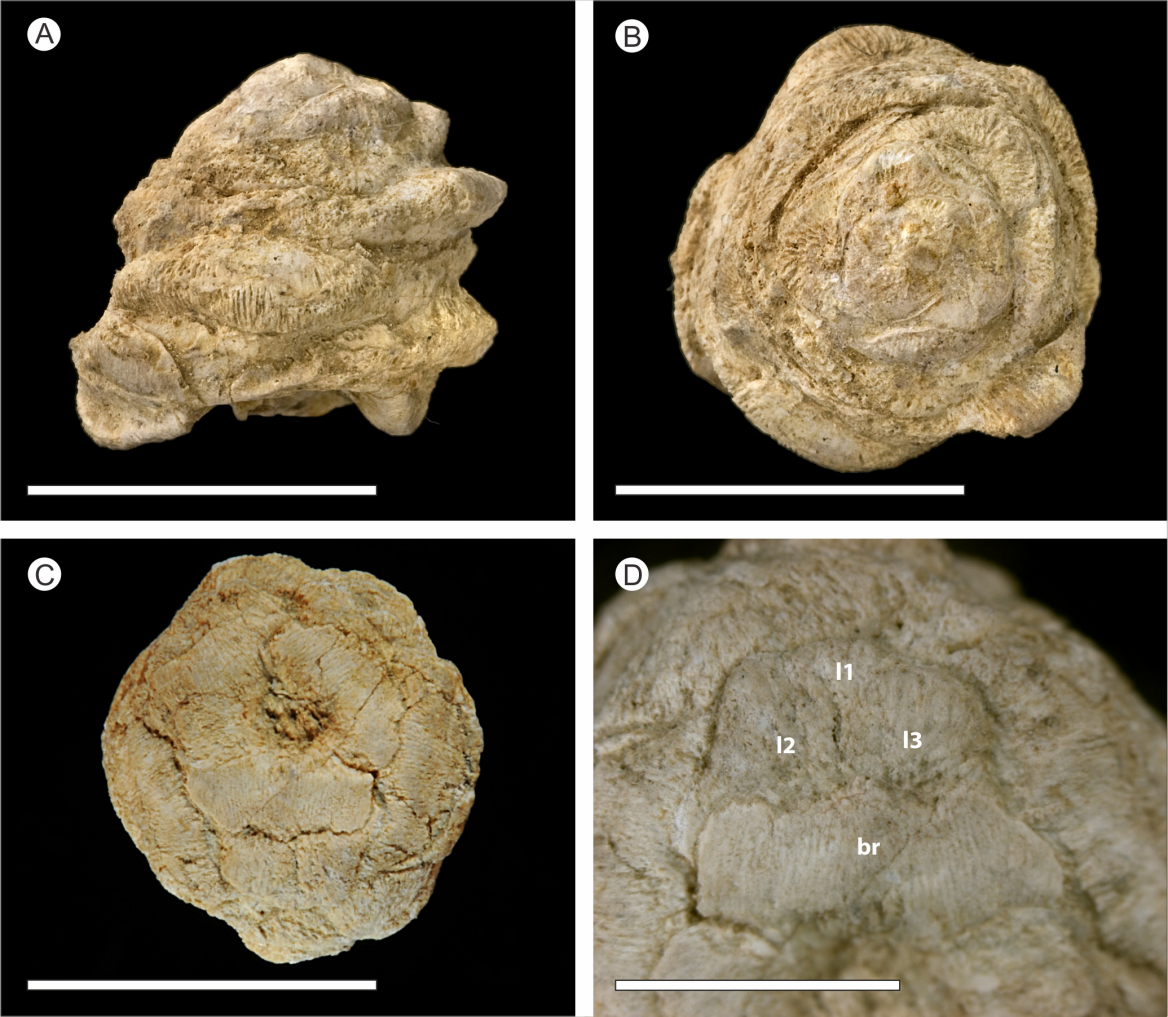
Photographed external morphology of cones. (A–B) Uncompressed cone NHMUK V 68524; (C–D) Compressed cone NHMUK V 68525. (A) Side view of cone; (B) Cone apex; (C) Base of compressed cone showing lobed ovuliferous scale; (D) Detail of ovuliferous scale, showing three lobes (L1 to L3) and bract (br). Scale bars for (A–C) = 1 cm, Scale bars for (D) = 0.5 cm.

**Figure 4:**
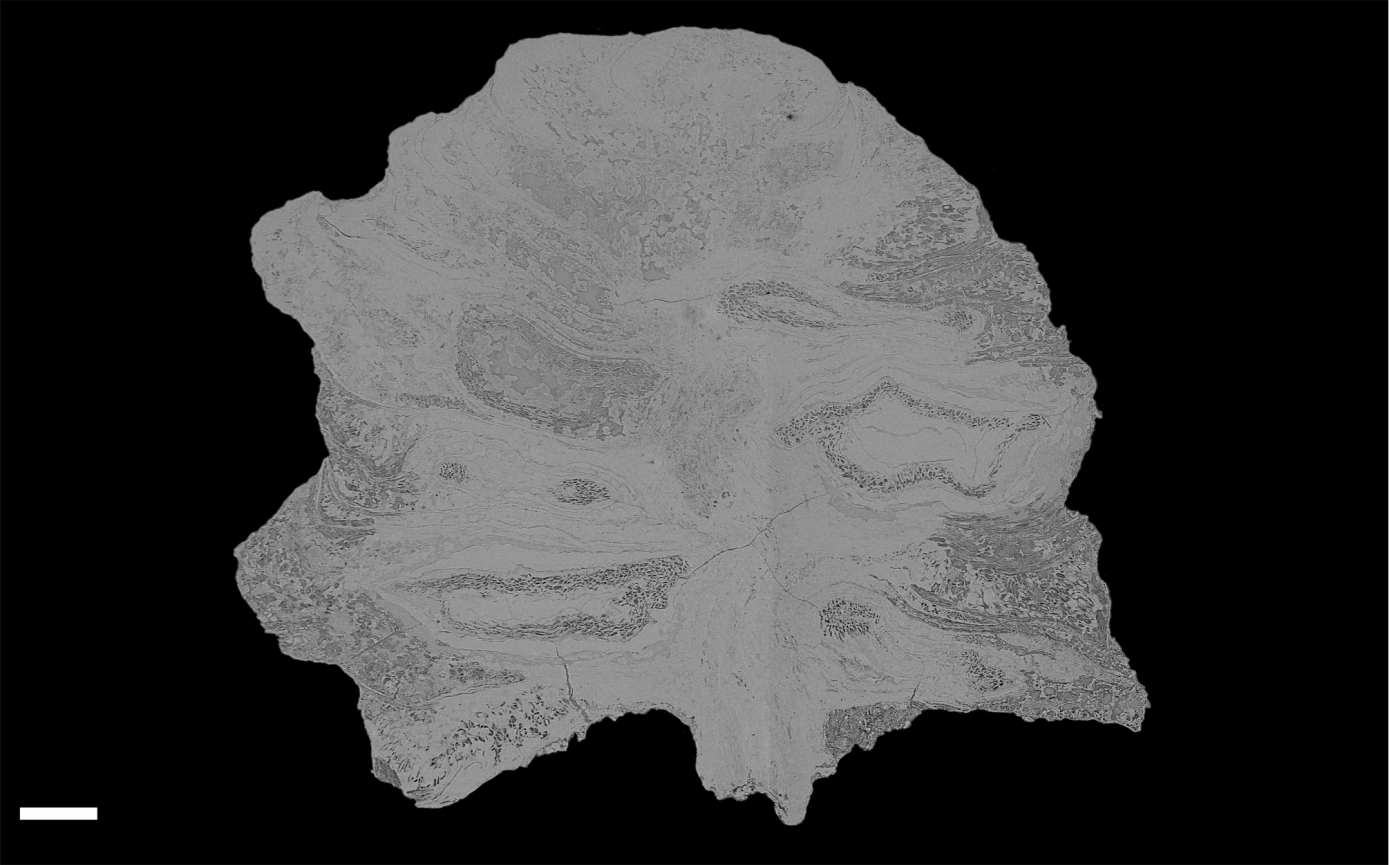
DLS synchrotron data showing gross morphology and anatomy of cone NHMUK V 68524. Image is depth merged to 15 µm. Longitudinal section showing whole cone. Scale Bar = 1 mm.

*Holotype hic designatus*. Natural History Museum (London) palaeobotanical collection number NHMUK V 68524 (Figs. 3A–3B, 4, 6, 9 and 10).

*Other material*. NHMUK V 68525.

*Etymology*. The specific epithet *collinsonae* is in honour of Professor Margaret E. Collinson for her contributions to the field of palaeobotany and palaeontology in general.

*Remarks*. The following combination of characters distinguish *P*. *collinsonae* from other species: cone shape spherical to ovoid (length:width approximately 1:1); small size of cone (ca. 1.24 cm long, 1.3 cm wide) and ovule (3–4 mm length and width); one ovule per bract scale complex; bract trace circular at origin; bract and scale traces that diverge from the cone axis separately; and absence of a layer ‘I’ beam cells (*sensu* Escapa et al., 2012; Escapa et al., 2013; Stockey & Rothwell, 2013, where the cells have an ‘I’-shaped cross section, similar to the iron or steel beams used in the construction industry) in the integument of the ovule (see Table 1).

**Table 1:** Morphological comparison of known *Pararaucaria* ovuliferous cones. After Stockey & Rothwell (2013).

Taxon	*Pararaucaria* *patagonica*	*Pararaucaria* *delfueyoi*	*Pararaucaria* *carrii*	*Pararaucaria* *collinsonae*	*Pararaucaria sp.*
**Age**	Middle Jurassic	Upper Jurassic	Middle Jurassic	Upper Jurassic	Upper Jurassic
**Distribution**	Santa Cruz, Argentina	Chubut, Argentina	Oregon, USA	Chicksgrove, UK	Utah, USA
**Length (cm)**	2.3–5.1	8.0	At least 2.8	1.24^*^	1.8–3.0
**Width (cm)**	1.3-3.0	3.0-4.0	At Least 1.3	1.3	1.7–2.0
**Cone Shape**	Cylindrical/Conical/ Ovoid	Cylindrical	Cylindrical	Spherical/Ovoid	Ovoid/obovoid to ellipsoid/oblong
**Number of Ovuliferous** **Scale adaxial lobes**	1 central 2 lateral	?	?	1 central 2 lateral	?
**No. seeds per scale**	1 (2^**^)	2	1	1	2
**Ovule/Seed Length (mm)**	6.0	11.0	5.3	3–4	5
**Ovule/Seed width (mm)**	6.0 (single seed), 3.3 (double seed)	5.0 (double seeds)	6.6	3–4	2.5
**Bract trace at origin (x.s.)**	Circular	Circular or subcircular	Crescent shaped	Circular	?
**Branching of bract trace**	?	?	Repeatedly	Repeatedly	?
**Bract transfusion tissue**	?	?	Present	Present	?
**Branching of scale trace**	Distally producing several strands	?	Vertically (seed bundle), distally producing several strands	Distally producing several strands	?
**Pocket forming tissue**	Parenchyma and anastomising branched cells	Parenchyma and anastomising branched cells	Parenchyma and numerous sclereids	Parenchyma and elongate sclereids	Present. Not described.
**Seed** **vascularization**	?	?	Cup shaped at base of nucellus	Vascular pad at base of nucellus	?
**References**	Wieland, 1935; Stockey, 1977; Escapa et al., 2012	Escapa et al., 2013	Stockey & Rothwell, 2013	Current report	Gee et al., 2014

**Notes.**

* cone length based upon uncompressed cone, which has the basal most bract/scale complexes missing. The missing bract/scale complexes are in the uncompressed cone are thought to be few in number, and the resultant shape of a complete cone is interpreted as being more or less as long as it is wide.

** only found in one occurrence where a cone with complexes containing two ovules each found on only one side of the cone was reported (GW Rothwell, pers. comm., 2014).

## Description

**Figure 5:**
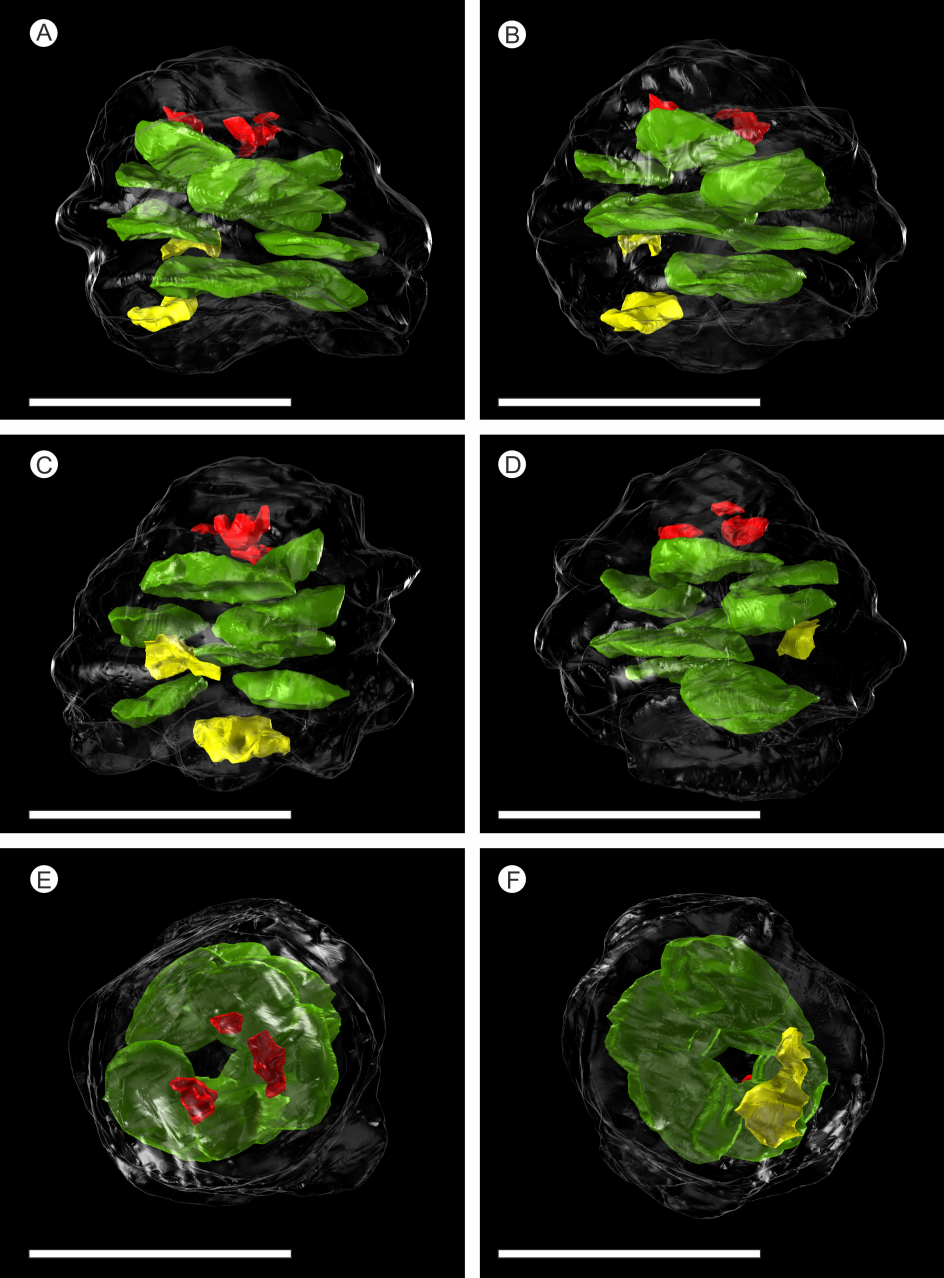
SPIERS 3D reconstruction of whole cone NHMUK V 68524, showing position of ovules. (A–D) Side of cone; (E) Cone apex; (F) Cone base. All scale bars = 1 cm. Key: transparent white outline, cone exterior; green, un-degraded ovules; red, immature ovules; yellow, degraded ovules.

**Figure 6:**
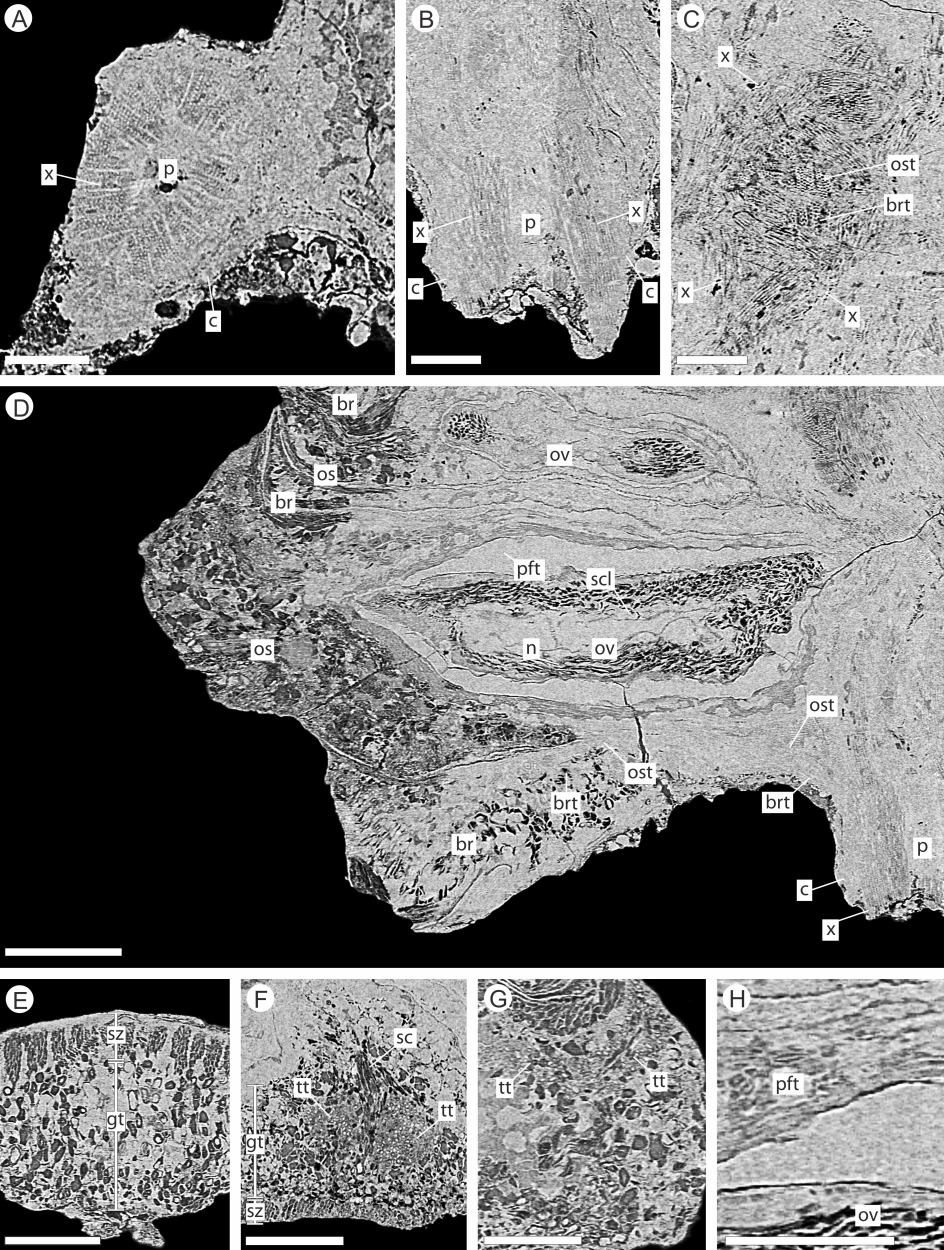
DLS synchrotron data showing internal anatomy of the *Pararaucaria collinsonae* cone NHMUK V 68524. (A) Cross-section of cone axis. Scale Bar = 0.5 mm; (B) Longitudinal section of cone axis. Scale Bar = 0.5 mm; (C) Longitudinal section through cone axis showing branching patterns causing by the attachment of a bract/scale complex. Scale Bar = 0.5 mm; (D) Longitudinal section through bract/scale complex. Scale Bar = 1 mm; (E) Cross-section through distal end of bract showing ground tissue and striation causing sclereid zones. Scale Bar = 0.5 mm; (F) Transverse section through bract showing histology just below the level the ovuliferous scale trace exits the bract into the scale. The sclerenchyma bundle that accompanies the vascular traces extends towards the margins of the bract with two zones of transfusion tissue either side. Scale Bar = 1 mm; (G) Longitudinal section through distal scale showing cellular anatomy. A zone of transfusion tissue with small vascular trace is seen extending towards the scale tip (not present due to abrasion). Scale Bar = 1 mm; (H) View of pocket-forming tissue enclosing the ovule. Scale Bar = 0.5 mm. Legend: c, cortex; br, bract; brt, bract trace; gt, ground tissue; n, nucellus; os, ovuliferous scale; ost, ovuliferous scale trace; p, pith; pft, pocket-forming tissue; ov, ovule; sar, sarcotesta; sc, sclerids/sclerenchyma; sz, sclereid zone; scl, sclerotesta; tt, transfusion tissue zone; x, xylem.

**Figure 7:**
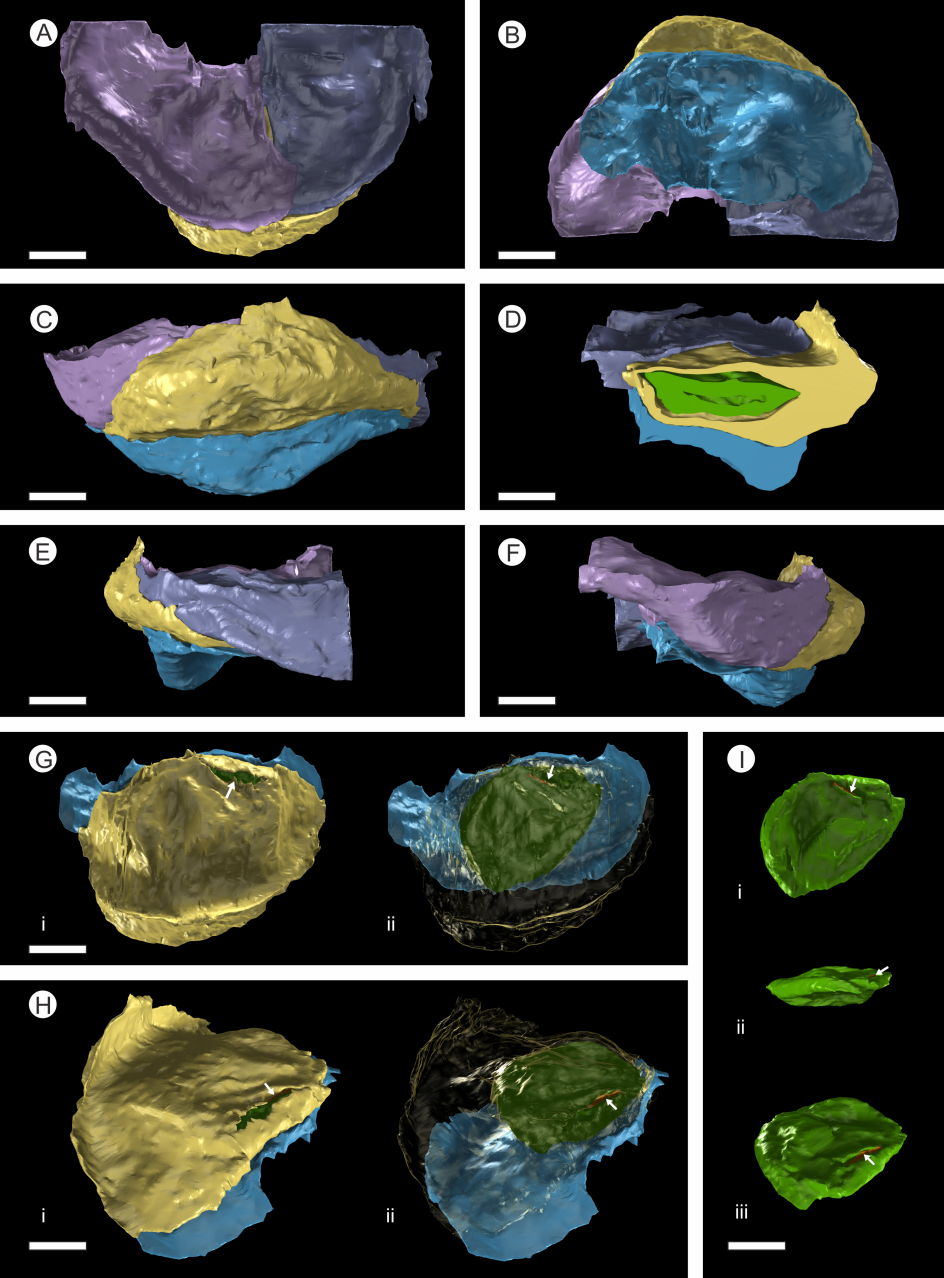
3D Model from DLS synchrotron x-ray dataset showing high resolution reconstruction of a bract/scale complex constrained by overlying bracts. (A) Top view of bract/scale complex; (B) Basal view of bract/scale complex; (C) Frontal view of bract/scale complex; (D) Virtual slice through bract/scale complexes showing internal morphological relationships; (E) Right-hand side view of bract/scale complex; (F) Left-hand side view of bract/scale complex; (G) Top view of bract/scale complex with overlying bracts removed (i), ovule position and micropylar region (see arrow) seen through transparent scale (ii); (H) Tilted side view of bract/scale complex with overlying bracts removed (i), ovule position and micropylar region (see arrow) seen through transparent scale (ii); (I) View of ovule and position of micropylar region (see arrow) seen in three views: (i) top, (ii) side, and (iii) tilted side. All Scale Bars = 1 mm. Key: blue, bract of bract/scale complex; yellow, ovuliferous scale of bract/scale complex; purple/mauve, overlaying bracts; green, ovule; orange, micropylar region.

The description herein is based on the exposed external surface morphology of both cones (Fig. 3), and X-ray imagery (Figs. 4, 6, 9 and 10) in the form of SRXMT tomographic data. This shows the cellular histology, forming the basis for 3D reconstructions of the internal morphology and anatomy of the uncompressed specimen NHMUK V 68524 (Figs. 5 and 7; Videos S1 and S2). Any colour descriptions used refer either to the visual colouration of the external surface of the cone or to the attenuation differences in the X-ray imagery within the specimen.

### External morphology

The cone is more or less spheroidal in vertical section with a concave base (Figs. 3A, 4 and 5A–5D) and is broadly circular in transverse section (Figs. 3B–3C, 4 and 5E–5F). It measures 1.23–1.30 cm at maximum diameter, is 1.24 cm in height, and is permineralized in silica with a pale white to cream colouration, the outer surface having been abraded, both pre- and post-silicification. The abrasion has not worn away the outer surface of the cone sufficiently to expose the ovules, though most of the surface ornamentation has been removed. In places the outer surface of the ovuliferous scale appears striated (Figs. 3A–3B). No laminar tip or umbo (*sensu* Escapa et al., 2012) has been observed.

### Cone axis

The cone axis is a woody cylinder between 1.0–1.4 mm in diameter with a small pith 0.3–0.6 mm in diameter (Figs. 6A–6B). The histology of the pith is not visible and may indicate that it was not preserved. The surrounding wood consists of radial rows of tracheids interspersed with rays (Figs. 6A–6B). No resin ducts or growth rings are visible. An amorphous layer surrounding the xylem may represent a zone of cambium, secondary phloem, and cortex (Figs. 6A–6B). The anatomy of the axis becomes increasingly convoluted in and around the area of bract/scale attachment (Fig. 6C). At this point, the bract/scale complex vascular traces can be seen to diverge from the main axis as two distinct bundles (Fig. 6C).

### Bract/scale complexes

The bracts and ovuliferous scales are helically arranged, with 3 helices in the clockwise direction and two helices in the counter-clockwise direction (Fig. 5; Videos S1 and S2). Individual bract/scale complexes have an exterior dorsiventrally flattened rhomboidal shape (Fig. 3), possess a maximum width of 8.0–9.0 mm and thickness of 1.5–2.0 mm at the cone base, and become progressively reduced in size towards the apex of the cone (Fig. 4). Their outer surface ornament has been eroded and cannot be determined with reliability over the whole cone (Fig. 3).

**Figure 8:**
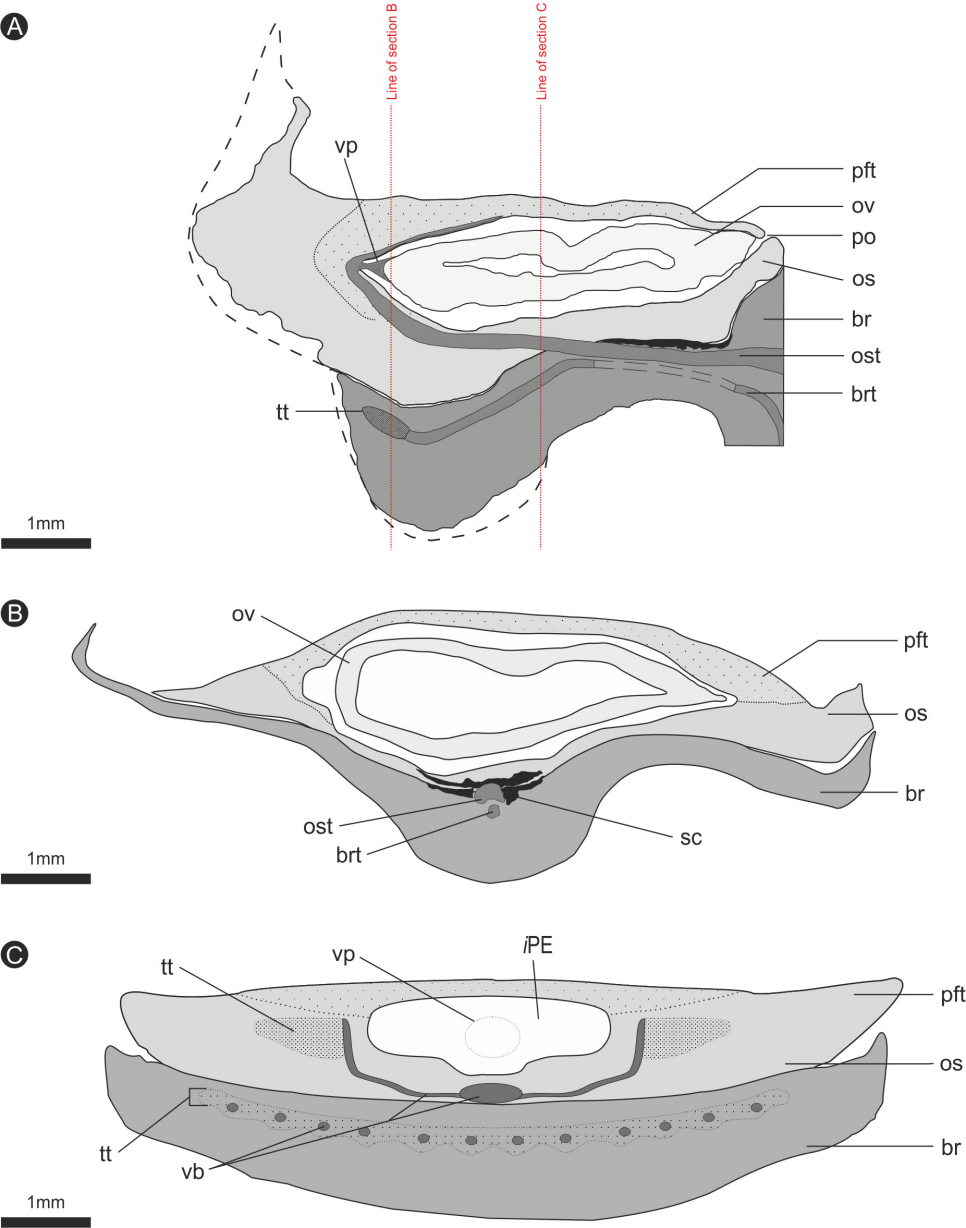
Composite diagrammatic sections showing key features of bract/scale complex. (A) longitudinal section through bract/scale complex; (B) cross-section through mid-point of bract/scale complex (see ‘A’ for line of section); (C) cross-section at point of ovule attachment within bract/scale complex (see ‘A’ for line of section). ‘Dashed line’, interpreted original outline. All Scale Bars = 1 mm. Legend: br, bract; brt, bract trace; iPE, internal surface of pocket forming tissue; os, ovuliferous scale; ost, ovuliferous scale trace; pft, pocket-forming tissue; po, pollen opening; ov, ovule; sc, sclerids/sclerenchyma; tt, transfusion tissue; vb, vascular bundles; vp, vascular attachment pad.

**Figure 9:**
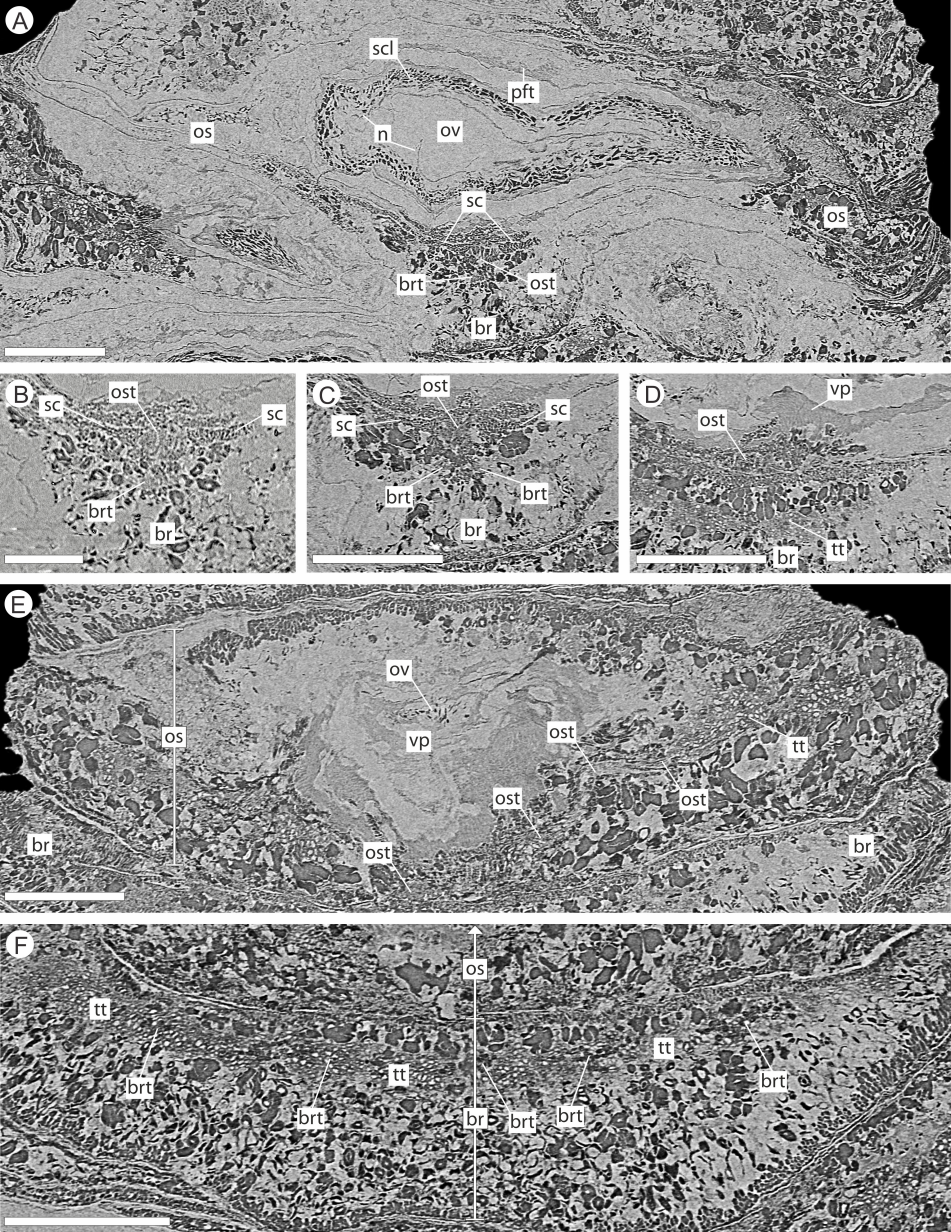
DLS synchrotron data showing internal anatomy of the *Pararaucaria collinsonae* cone NHMUK V 68524. (A) Transverse section through bract/scale complex, showing position of bract and scale trace. Note the two triangular sclerenchyma strands that accompany the scale trace through the cortex of the cone axis. Scale Bar = 1 mm; (B) Transverse section through bract/scale axis near cone axis showing cellular anatomy. Scale Bar = 0.5 mm; (C) Tangential section through bract/scale axis near scale separation point, showing bract-trace commencing to split horizontally. Scale bar = 1 mm; (D) Tangential section through bract/scale showing the start of the zone of transfer tissue within bract. Scale Bar = 1 mm; (E) Tangential section through bract/scale axis showing vascularization of bract and cellular structure. Scale Bar = 1 mm; (F) Tangential section through bract/scale axis showing vascularization of bract and cellular structure. Scale Bar = 1 mm. Images (A), (C–F) are depth merged to 15 µm, B to 5.4 µm. Legend: br, bract; brt, bract trace; n, nucellus; os, ovuliferous scale; ost, ovuliferous scale trace; pft, pocket-forming tissue; ov, ovule; sc, sclerids/sclerenchyma; tt, transfusion tissue; vp, vascular attachment pad.

The bract/scale complex consists of a large three-lobed ovuliferous scale (Figs. 3C–3D) that is subtended by a broad bract (Fig. 6D; Videos S1 and S2). The ovuliferous scales are generally eroded to the same length as the bract, with the exception of one scale at the base of the cone (Figs. 3C–3D), and have a maximum observed width of 8.7 mm, 1.3 mm high at cone axis, increasing to 2.3 mm medially and 5.0 mm at the tip (Figs. 7A–7F). The bract and scale are attached basally in a zone ca. 0.8–1.1 mm wide to ca. 1.4 mm distal from the cone axis. The bract has a ‘gull wing’ cross-sectional shape, having a V-shaped central axis with lateral ‘wings’. The bract is 0.9–1.0 mm thick at the cone axis thickening to at least 1.3–1.7 mm and 4.0 mm long distally, the wings thin to 0.1 mm at the margins (Figs. 7A–7F and 8A–8C). Distally, the bract produces a rim. At the centre the height is 0.6 mm with an acute angle of 40° from horizontal, and towards the lateral margins the height raises to 0.7 mm with an acute angle of 80° from horizontal (Figs. 7A–7F and 8A–8C). The extent and full dimension of the distal margins are not certain due to erosion. The ground tissue of the bract appears to mainly consist of larger thick-walled parenchyma with zones of elongate sclereids at the external surface (Figs. 7D–7F). Towards the distal end of the bract a zone of transfusion tissue, ca. 69 µm thick, extends as a layer above the ground tissue (Fig. 9F).

In longitudinal section, the ovuliferous scale tissue extends parallel to the upper surface of the abaxial bract, outwards from the cone axis and beyond the ovule, where it then arches back over the ovule to form an ovule-enclosing pocket (Figs. 6D; 7D, 7G–7H; 8A; 9A). The pocket-forming tissue is ca. 250 µm thick distally and thins proximally as it progresses towards the cone axis. It terminates prior to reaching the cone axis, leaving an adaxial slit-like opening exposing the micropylar region (Figs. 6D, 7D, 7G–7H and 8A). The histology of the pocket-forming tissue is poorly recovered using SRXMT with only a faint outline showing its extent. Distally, the pocket forming tissue appears to be predominantly parenchymatous with occasional possible elongate sclereids (Fig. 6H). The X-ray contrast is poor at its upper and lower margins, but the cellular features are quite distinct towards the interior, probably indicating that the outmost margins are less completely preserved than the interior. Medially and proximally towards the cone axis, the pocket forming tissue lacks sufficient X-ray contrast to comment upon the nature of the cell structure, though the tissue boundaries are quite clear (Fig. 6D).

The vascular traces to the bract and to the ovuliferous scale diverge from the stele as two separate bundles (Figs. 6C and 8A). The ovuliferous scale traces travel outwards horizontally from the stele at ca 90°, whilst the bract traces diverge from the stele asymptotically, curving upwards towards the scale trace. The bract trace then runs parallel with but below the ovuliferous scale trace for ca. one third of its course, before descending at ca. 30° into the distal region of the bract, where it fans out horizontally, vascularizing a transfusion tissue zone. The scale-trace at the bract mid-point (Figs. 9A–9B) forms a 379 × 90 µm ‘U’ with a layer of sclerenchyma tissue above and a pair of separate triangular sclerenchymatous bundles to either side (Fig. 9A). The bract-trace, round in shape and measuring 54 × 96 µm, is located below the scale-trace and is separated from it by a thin layer of parenchyma cells (Figs. 9A–9B). Where the scale-trace exits the bract into the scale, it is more rounded in shape, with dimensions of 252 × 168 µm, the two triangular sclerenchyma bundles continue to constrain the trace either side (Figs. 9A–9C). Towards the distal end of the ovule, the bundle branches into a fan of smaller vascular traces which rise up vertically forming the vascular pad for the ovule, at this point the bundle divides into two horizontal bundles that extend horizontally and vertically around the outer edge of the ovule cavity (Fig. 9E). The finial number of vascular bundles and their exact course could not be determined due to poor X-ray contrast and erosion of the scale apices. The bract-trace diverges horizontally at the point where the scale-trace enters the scale into multiple distinct bundles with average dimensions at this point of 90 × 90 µm (Fig. 9C). These bundles subsequently diverge horizontally further along the line of the bract parenchymatous transfusion tissue (Fig. 9F).

### Ovules

There are fourteen ovules in various states of preservation and development, of which nine are well preserved and of similar size with in the cone (Figs. 5A–5F, Video S1). At the cone apex the three topmost ovules are poorly preserved, and greatly reduced in size, the uppermost of these being notably smaller (Figs. 5A–5F). These three ovules are almost certainly immature or abortive due to their proximity to the cone apex. The following nine well preserved ovules are flattened ellipsoidal disks, truncate to rounded at the micropylar end and roundly tapering towards the chalaza. In cross section, the ovules are ovately flattened (Figs. 7G–7I, 8A–8B and 9A). There is a single inverted ovule on each ovuliferous scale, and the mature well preserved ovules vary between 3–4 mm wide, 3–4 mm long and ca. 2 mm thick. There is evidence that the ovules have shrunk or collapsed away from the enclosing pocket forming tissue during diagenesis in that there is a non-cellular infill matrix between the pocket forming tissue and the outer integument of the ovule, and that the integument forms a sinuous and occasionally convoluted margin (Figs. 4, 6D and 8A–8B). The three dimensional model shows that ovules are inverted, with the centre line of the ovules being ca. 40° offset from the centre line of the seed/scale complex. The micropylar region, an elongate depression (Figs. 7G–7I), faces towards the cone axis with an offset the same as the ovule and with the chalaza distal from the cone axis (Fig. 7G). The ovules are attached at the chalaza to the inner surface of the scale tissue (Fig. 8A). The two basal most ovules are poorly preserved, preventing an analysis of their morphology.

**Figure 10:**
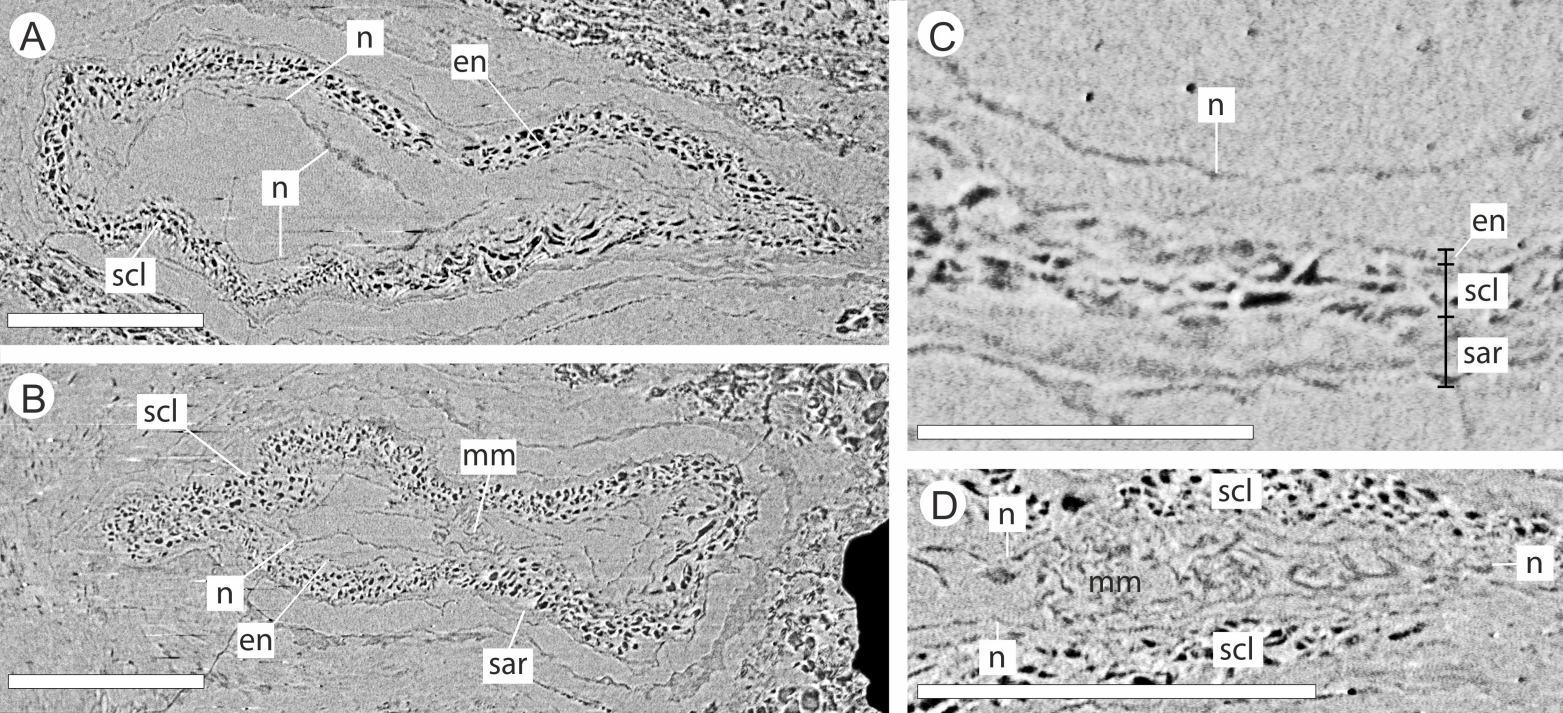
DLS synchrotron data showing cellular anatomy of ovules of *Pararaucaria collinsonae*. (A) longitudinal section through ovule; (B) cross-sectional view through ovule including the megagametophyte membrane; (C) view of ovule integument construction, showing the nucellus free of the endotesta within the ovule; (D) view of convoluted megagametophyte membrane within the nucellus. Scale Bar (A), (B) & (D) = 1 mm; (C) = 0.5 mm. Legend: en, endotesta; mm, megagametophyte membrane; n, nucellus; sar, sarcotesta; scl, sclerotesta.

The integument has multiple layers. The first of these consists of an outer zone of poorly defined outer sarcotesta that is 50–84 µm wide (Figs. 10A–10C), followed by a thicker (ca. 45–182 µm) middle layer, with dark cells whose lumens follow sinuous paths in general alignment with the ovule axis (Figs. 10A–10D). In places this layer of cells becomes more convoluted and less well preserved. The final integumentary layer is a thin, cellular monolayer or endotesta, 10–13 µm wide consisting of small ovate cells (Figs. 10A–10C).

The nucellus, where preserved, is 5–10 µm wide, convoluted, and collapsed within the integumentary hollow, and does not appear to be adnate to the integument (Figs. 10A–10D). The megaspore membrane, where visible, is 15 µm thick and appears to form a cellular monolayer. No megagametophyte tissue within the membrane can be discerned (Fig. 10D).

## Discussion

### Systematic relationship

An affinity of *P. collinsonae* with *Pararaucaria* was first suggested by Needham (2011) and is here confirmed based on cone anatomy and morphology. The cones of Cheirolepidiaceae can be identified through the presence of distal lobes on the ovuliferous scales. Because the exteriors of permineralized cones are frequently abraded, however, this feature is not always preserved (Escapa et al., 2013). In our two cones, abrasion has removed the scale tips on all but one of the bract/scale complexes, where three distal lobes are present. Of the internal features supporting an affinity with Cheirolepidiaceae, the presence of adaxial pocket-forming tissue is most diagnostic, forming a distinctive flap that overarches and encloses the ovules on the adaxial side of the ovuliferous scale (Escapa et al., 2012). In our uncompressed cone, pocket-forming tissue is clearly discernable in radial longitudinal section of all mature ovuliferous scales. In summary, *Pararaucaria* (Wieland, 1929; Wieland, 1935; Stockey, 1977; Escapa et al., 2012; Escapa et al., 2013; Stockey & Rothwell, 2013) affinities are based on the following shared characters: (1) cylindrical/ovoid cone shape; (2) helically arranged bract/scale complexes; (3) ovuliferous scale and bract unfused except for a small zone at the bract mid-point; (4) independent bract/scale traces for much of their length; (5) inverted ‘U’-shaped vascular scale trace; and (6) bract which is broader than the ovuliferous scale. This combination of features distinguish *Pararaucaria* from other cone genera within the Cheirolepidiaceae (Escapa et al., 2012; Escapa et al., 2013; Stockey & Rothwell, 2013).

Previous studies on *Pararaucaria* have identified three species, and one morphotype, namely *P. patagonica* Wieland (Middle Jurassic Cerro Cuadrado Petrified Forest, Patagonia, Argentina) (Wieland, 1935; Stockey, 1977; Escapa et al., 2012), *P. delfueyoi* (Escapa et al., 2013) (Upper Jurassic Cañadón Calcáreo Formation, Chubut Province, Argentina), *P. carrii* (Stockey & Rothwell, 2013) (Middle Jurassic Trowbridge Formation, near Izee, Oregon, USA), and an unnamed morphotype from the Morrison Formation (Upper Jurassic, Utah, USA; Gee et al., 2014). *P. collinsonae* most closely resembles *P. patagonica* and *P. carrii*. Similarities include: (1) the triangular shape in cross-section of the sclerenchyma bundles accompanying the ovuliferous scale trace; (2) the multi-layered integument; (3) one ovule per bract/scale complex; and (4) the specialised pad of tissue attaching the ovules at the chalaza (Stockey, 1977; Escapa et al., 2012; Stockey & Rothwell, 2013). The sclerenchyma bundles that accompany the ovuliferous scale trace in the Chicksgrove cone are more gracile than those of other species. The Chicksgrove cone also possesses a fan of transfusion tissue in the bract, similar to that figured for *P. carrii* (Stockey & Rothwell, 2013). This feature was not noted in the other species (Escapa et al., 2012; Stockey & Rothwell, 2013). Further similarities to *P. patagonica* include an external scale surface with longitudinal striations caused by the sub-surficial cellular pattern (Stockey, 1977; Escapa et al., 2012) and a three-lobed ovuliferous scale Escapa et al. (2012). In accordance with the other known species of *Pararaucaria*, there is no evidence of resin canals in the wood or ground tissues of the Chicksgrove cone (Escapa et al., 2012; Escapa et al., 2013; Stockey & Rothwell, 2013).

*Pararaucaria collinsonae* can be readily distinguished from other *Pararaucaria* species on the basis of the number of ovules per ovuliferous scale, on cone size and shape, and on ovule size (Table 1). *P. delfueyoi* and the Morrison Formation morphotype is distinguished from congeneric species by the occurrence of two ovules per bract/scale complex (Wieland, 1935; Stockey, 1977; Gee et al., 2014). The Chicksgrove cone, like *P. carrii* and the majority of *P. patagonica*, developed only one ovule per bract/scale complex. *P. collinsonae* is also smaller than the other species: although similar in width to *P. carrii* and *P. patagonica*, it is at least 50% shorter than both, and significantly smaller than *P. delfueyoi* (Table 1). We believe this reflects its true size: although one cone is incomplete at the base, the second compressed cone shows evidence of shortening bracts basally, indicative of a more intact base. Both Chicksgrove cones are similar in diameter. Ovule size shows a similar pattern being at least 40% shorter and narrower than in any other species (Table 1). We consider it unlikely that this smaller ovule size represents an immature state due to the presence of a clear developmental series of ovules within the Chicksgrove cone. At least three immature ovules near the apex are followed by a series of 11 in the remainder of the cone (three of which are highly degraded). All are of similar dimensions, indicating that the large group of ovules were developmentally mature. It is also worth noting that although the Chicksgrove cone has a similar overall diameter to both *P. patagonica* and *P. carrii*, its ovules are significantly smaller. We thus conclude that the combination of size difference in cones and ovules constitutes a diagnostic character distinguishing the Chicksgrove cone from the other species of *Pararaucaria*.

There are several additional subtle points of morphological difference that separate the Chicksgrove cone from other species of *Pararaucaria*. The ovules are elliptical not cordate as in *P. patagonica* (Stockey, 1977; Escapa et al., 2012). There are differences in the cross-sectional outlines of the bract traces near their origin from the stele, which have been used to differentiate species (Stockey & Rothwell, 2013). Specifically, *P. collinsonae* resembles the *P. patagonica* state in having a circular cross-section, whereas in *P. delfueyoi* bract traces are subcircular, and in *P. carrii* they are crescent-shaped. Differences also exist between the integuments of the Chicksgrove cone and those of *P. patagonica*. The former lacks distinctive zone of cell layers that superficially resemble ‘I-beams’ in *P. patagonica* ovules (Escapa et al., 2012). It should be noted, however, that ‘I-beam’ cells might be a preservational effect rather than a natural feature of the ovules. In summary, these differences in overall morphology and internal structure justify the erection of a new species.

### Synchrotron tomography

Synchrotron X-ray Microtomography (SRXMT) is now widely applied in palaeobotanical research. The method has been used to image a variety of small mineralised (calcite, pyrite) and non-mineralised (charcoal, coal) plant fossils. SRXMT has been applied to calcified Palaeozoic charophyte oogonia (Feist, Lui & Tafforeau, 2005), minute charcoalified flowers from the Early Cretaceous (Friis et al., 2007), charcoalified pteridosperm ovules and pollen organs acid macerated from Lower Carboniferous limestones (Scott et al., 2009), Carboniferous (Glasspool et al., 2009) and Permian (Slater, McLoughlin & Hilton, 2011) megaspores, coalified fruits and seeds preserved in a Middle Eocene oil shale (Collinson et al., 2012), and the earliest wood permineralized in pyrite from sediments of Early Devonian age (Strullu-Derrien et al., 2014). This study represents the first application of SRXMT to plant fossils permineralized in silica. Previous studies all used micro-sized (<5 mm) specimens or targeted area-of-interest scanning due to the limited penetration power and/or cross-sectional area of the beam. The DLS I12 beamline has the advantage of a large beam cross-sectional area and high X-ray energy and high brilliance. This enabled us to completely image a plant fossil whose volume (ca. 1.2 cm^3^) is considerably larger than previously imaged plant fossils (ca. 0.1–0.125 cm^3^), opening up a new size class of fossil plant parts to investigation.

Third-generation synchrotrons are increasingly commonplace and accessible, and facilitate beamlines with hard monochromatic X-rays. Their high brilliance allows focussing optics, which in turn can yield very high resolution scans (e.g., 0.35 µm voxel size) in a shorter time, and with fewer technical challenges than in a lab-based setting. In addition, there are no beam-hardening artefacts, resulting in a more accurate map of X-ray attenuation within the specimen (Smith et al., 2009). Examination of the external surfaces of the scanned cone shows that it has some porosity, which is evident in the ribbing of the abraded bract/scale complexes. This indicates that some of the cells within the tissues—and perhaps also the tissue boundaries—might represent minute voids (i.e., dark areas in images). In others, cell lumens appear to be infilled with mineral (i.e., bright areas in images), and here the darker cell boundaries might reflect the presence of organics derived from the original cell walls. Some tissue systems appear to retain little cell structure (e.g., interiors of ovules) suggesting a more advanced state of decay prior to silicification. The lack of cellular detail in the ovule interiors may, however, be alternatively interpreted as the preservation of a pre-cellular stage of megagametophyte maturation as is typical of most fossil ovules (GW Rothwell, pers. comm., 2014). Clearly, SRXMT picks out the more robust tissue systems, including vascular tissues, sclerotesta and sclerenchyma. The level of resolution attainable is remarkable, but in our specimens it does not match that of high quality petrographic thin sections (e.g., Escapa et al., 2012; Stockey & Rothwell, 2013). This limitation should be weighed against the method’s great advantages such as its versatility, the three-dimensional picture of the organisation of internal tissue systems provided, and its non-destructive nature. The strength of SRXMT over XMT of similar silicified fossil cone materials (e.g., Gee, 2013; Gee et al., 2014) lies in its ability to resolve structure at the cellular level, enabling a more precise demarcation of tissue boundaries, and hence organ shape, and also a better assessment of the constituent nature of the tissues under investigation. This additional level of detail allows a more accurate appraisal of affinities.

### Purbeck forest and palaeobiogeography

*Pararaucaria collinsonae* extends our understanding of the dominant tree element of the Late Jurassic (Tithonian Stage) Purbeck Forest. This was a seasonally arid forest of conifers and cycadophytes (Bennettitales), which flourished around the margins of a shallow hypersaline gulf that extended over a large swathe of SE England and the English Channel (West, 1975; Francis, 1983; Francis, 1984; Bradshaw et al., 1992; Needham, 2011; Fig. 1). The fossilised remains are located in the Purbeck Group (Lulworth Formation; Cope, 2008), where they are most commonly encountered in small coastal exposures and during the quarrying of building stones, notably the Portland Stone (Falcon-Lang, 2011). The silicified tree stumps, roots, and fallen trunks and branches are especially well preserved in a thin palaeosol known as the Great Dirt Bed. By far the most common type of fossil wood is attributable to *Protocupressinoxylon purbeckensis* (Francis, 1983; Philippe & Bamford, 2008; Philippe et al., 2010). Based on a detailed study of this wood—together with foliage (*Cupressinocladus valdensis*), pollen cones (*Classostrobus* sp.), and pollen (*Classopollis* sp.) found as associated elements in some palaeosols—Francis (1983) concluded that the dominant forest tree was a conifer in the Cheirolepidiaceae. Our results add further weight to this conclusion and provide the first evidence of ovulate cheirolepideaceous cones from the Purbeck Forest. In overall size the ovulate cones are about the same length as the largest known Purbeck pollen cones of *Classostrobus* and about twice the width. They were collected from a new exposure of a thin palaeosol, which we tentatively equate with the Great Dirt Bed or Lower Dirt Bed. This is based on our stratigraphic log, the nature of the palaeosol, and its associated fossil plant remains. The cones were retrieved from fissures in the thrombolytic limestone underlying the palaeosol, where they were closely associated with silicified roots, an *in situ* tree stump, a fallen trunk and numerous branches. Although association alone cannot prove that the cones were produced by the trees at this site, in the context of a forest preserved *in situ* it provides strong circumstantial evidence.

**Figure 11:**
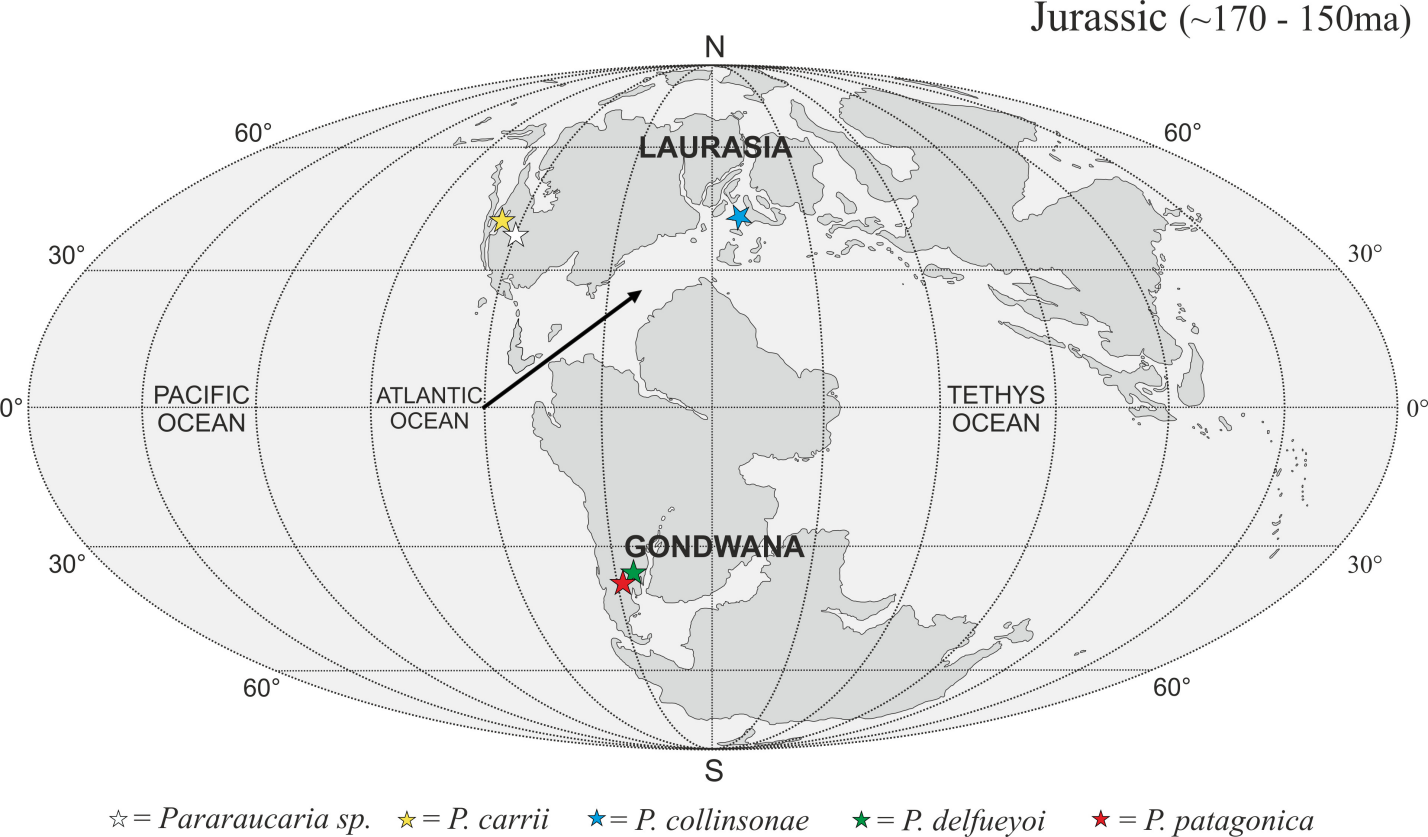
Global palaeogeographic map showing Jurassic (∼150–170 ma) locations of female *Pararaucarian* cones. Basemaps modelled upon Blakey (2008).

The *P. collinsonae* cones are the first evidence of *Pararaucaria* in Europe. The genus was originally established for permineralized ovulate cones from Argentina (see Escapa et al., 2012), but recently its known geographic distribution has expanded greatly to encompass sites in North America (Stockey & Rothwell, 2013; Gee et al., 2014). Together with the new data documented here, this translates to a mid-palaeolatitudinal distribution for *Pararaucaria* in both Gondwana and Laurasia during the Late Jurassic (Fig. 11). Such a distribution is consistent with the general pattern observed for Cheirolepidiaceae cones throughout most of the Mesozoic (see Data S1). Although based on records from only a handful of sites, this distribution might reflect some general trends in Mesozoic floras and climates. During the Late Jurassic, plant diversity and biome productivity differed markedly to the modern world. Today, tropical rainforest is the most diverse and productive ecosystem, but there is no evidence for this biome during the Late Jurassic (Sellwood & Valdes, 2008). Indeed, the General Circulation Models (GCMs) and biome reconstructions for this period indicate a largely arid to semi-arid low latitude region (Rees et al., 2004; Sellwood & Valdes, 2008). Furthermore, analysis of the taxonomic diversity and palaeogeographic distributions of fossil floras, combined with an assessment of the global distributions of key environmentally sensitive sediments (e.g., evaporates, calcretes, coals), and predictions from GCMs of climate, indicate that plant diversity was greatest at mid latitudes. They also suggest that this phenomenon was related to the very different climatic regimes that prevailed at the time (Rees, Ziegler & Valdes, 2000; Rees et al., 2004; Sellwood & Valdes, 2008). Evaporite deposits were mostly restricted to latitudes of between 40°N and 40°S where floras were of low diversity with high proportions of xerophytic microphyllous conifers, cycadophytes, pteridosperms, sphenophytes and ferns (Rees, Ziegler & Valdes, 2000; Rees et al., 2004). Coals became more abundant at mid to high-latitudes, where floras possessed more macrophyllous species and a higher stature vegetation (Rees et al., 2004). Ginkgophytes and macrophyllous conifers dominated in the highest latitude polar floras. The records of *Pararaucaria* fall within the higher latitude northern and southern limits of the distribution of evaporites, and lower latitude limits of coal deposits indicating an adaptation to semi-arid climates (Rees et al., 2004). GCMs place *Pararaucaria* within the ‘warm mixed forest’ or ‘temperate conifer forest’ biomes (Sellwood & Valdes, 2008). Models reconstruct the climates for these zones as having cool to mild winters (seasonal air temperatures of ca. 12 °C) and warm to hot summers, with the southern *Pararaucaria* experiencing very hot (>30–34 °C) summers. Palaeo-precipitation estimates indicate that *Pararaucaria* was adapted to semi-arid sites whose daily rainfall probably did not exceed 4 mm for any significant part of the year, but which had >1 mm or more during the driest part of the year. This is consistent with the palaeoenvironmental interpretation of the Purbeck Forest derived from tree distributions, tree ring analysis and associated climate sensitive sediments, which indicates that conditions for tree growth were marginal and that the climate was of Mediterranean type (Francis, 1984). This picture emerging for *Pararaucaria* therefore is one of trees that were elements of low diversity floras of semi-arid to Mediterranean environments.

### Conclusions

1. A new Upper Jurassic Cheirolepidiaceae ovuliferous cone species of *Pararaucaria* (*P. collinsonae)* has been described.
2. The anatomy, morphology and histology of a permineralized ovuliferous cone was elucidated using SRXMT for the first time.
3. The palaeogeographical occurrence of the genus *Pararaucaria* has been extended into Eurasia.
4. The global distribution of the *Pararaucaria* conforms to the same temperate zone pattern seen in the distribution of previously published Cheirolepidiaceae reproductive organs.
5. It was noted that most of the published Cheirolepidiaceae cone floras are clustered (especially in Northern Europe), emphasizing the need to reduce sampling bias by further collection in new and diverse continental settings.

## Supplemental Information

10.7717/peerj.624/supp-1 Data S1 Palaeogeographical Data based on literature survey. This file contains the results, dataset, and references from the literature survey.

10.7717/peerj.624/supp-2 Video S1 Video of 3D reconstruction showing gross morphology. Whole cone shown with ovule positions in 3D, followed by detailed views of a single bract/scale complex in 3D.

10.7717/peerj.624/supp-3 Video S2 Video of 3D reconstruction showing animated virtual sectioning through a single bract/scale complex.

## Additional Information and Declarations

### New Species Registration

The following information was supplied regarding the registration of a newly described species: The Natural History Museums K-Emu system was used to register our two specimens. The museum registry numbers were NHMUK V 68524 and NHMUK V 68525.

## References

[ref-1] Abràmoff MD, Magalhães PJ, Ram SJ (2004). Image processing with ImageJ. Biophotonics International.

[ref-2] Alvin K (1982). Cheirolepidiaceae: biology, structure and paleoecology. Review of Palaeobotany and Palynology.

[ref-3] Archangelsky S (1968). On the genus *Tomaxellia* (Coniferae) from the Lower Cretaceous of Patagonia (Argentina) and its male and female cones. Botanical Journal of the Linnean Society.

[ref-4] Axsmith BJ, Jacobs BF (2005). The conifer *Frenelopsis ramosissima* (Cheirolepidiaceae) in the Lower Cretaceous of Texas: Systematic, biogeographical, and paleoecological implications. International Journal of Plant Sciences.

[ref-5] Batten DJ (1974). Wealden Palaeoecology from the distribution of plant fossils. Proceedings of the Geologists’ Association.

[ref-6] Benton MJ, Cook E, Hooker JJ (2005). Mesozoic and tertiary fossil mammals and birds of Great Britain.

[ref-7] Blakey RC (2008). Gondwana paleogeography from assembly to breakup—a 500 m.y. odyssey. Geological Society of America Special Papers.

[ref-8] Bradshaw MJ, Cope JCW, Cripps DW, Donovan DT, Howarth MK, Rawson PF, West IM, Wimbledon WA, Cope JCW, Ingham JK, Rawson PF (1992). Jurassic. Atlas of palaeogeography and lithofacies.

[ref-9] Calder MG (1953). A coniferous petrified forest in Patagonia. Bulletin of the British Museum (Natural History) Geology.

[ref-10] Clement-Westerhof JA, Van Konijnenburg-Van Cittert JHA (1991). *Hirmeriella muensteri*: new data on the fertile organs leading to a revised concept of the Cheirolepidiaeae. Review of Palaeobotany and Palynology.

[ref-11] Coiffard C, Gomez B, Kvaček J, Thevenard F (2006). Early angiosperm ecology: evidence from the Albian-Cenomanian of Europe. Annals of Botany.

[ref-12] Collinson ME, Smith SY, Manchester SR, Wilde V, Howard LE, Robson BE, Ford DF, Marone F, Fife JL, Stampanoni M (2012). The value of X-ray approaches in the study of the Messel fruit and seed flora. Palaeobiodiversity and Palaeoenvironments.

[ref-13] Cope JCW (2008). Drawing the line; the history of the Jurassic-Cretaceous boundary. Proceedings of the Geologists’ Association.

[ref-14] Del Fueyo GM, Archangelsky S, Llorens M, Cuneo R (2008). Coniferous ovulate cones from the Lower Cretaceous of Santa Cruz Province, Argentina. International Journal of Plant Sciences.

[ref-15] DeVore ML, Kenrick P, Pigg KB, Ketcham RA (2006). Utility of high resolution X-ray computed tomography (HRXCT) for paleobotanical studies: an example using London Clay fruits and seeds. American Journal of Botany.

[ref-16] Escapa IH, Cuneo NR, Rothwell G, Stockey RA (2013). *Pararaucaria delfueyoi* sp nov from the Late Jurassic Canadon Calcareo Formation, Chubut, Argentina: insights into the evolution of the Cheirolepidiaceae. International Journal of Plant Sciences.

[ref-17] Escapa IH, Rothwell GW, Stockey RA, Cuneo NR (2012). Seed cone anatomy of Cheirolepidiaceae (coniferales): reinterpreting *Pararaucaria patagonica* wieland. American Journal of Botany.

[ref-18] Falcon-Lang HJ (2011). The Isle of Portland, Dorset, England. Geology Today.

[ref-19] Feist M, Lui J, Tafforeau P (2005). New insights into Paleozoic charophyte morphology and phylogeny. American Journal of Botany.

[ref-20] Francis JE (1983). The dominant conifer of the Jurassic Purbeck Formation, England. Palaeontology.

[ref-21] Francis JE (1984). The seasonal environment of the Purbeck (Upper Jurassic) fossil forests. Palaeogeography, Palaeoclimatology, Palaeoecology.

[ref-22] Friis EM, Crane PR, Pedersen KR, Bengtson S, Donoghue PCJ, Grimm GW, Stampanoni M (2007). Phase-contrast X-ray microtomography links cretaceous seeds with Gnetales and Bennettitales. Nature.

[ref-23] Garwood RJ, Dunlop JA (2014). The walking dead: blender as a tool for palaeontologists. Journal of Palaeontology.

[ref-24] Gee CT (2013). Applying microCT and 3D Visualization to Jurassic Silicified Conifer Seed Cones: a virtual advantage over thin-sectioning. Applications in Plant Sciences.

[ref-25] Gee CT, Dayvault RD, Stockey RA, Tidwell WD (2014). Greater palaeobiodiversity in conifer seed cones in the Upper Jurassic Morrison Formation of Utah, USA. Palaeobiodiversity and Palaeoenvironments.

[ref-26] Glasspool IJ, Collinson ME, Scott AC, Brain APR, Plotnick RE, Kenig F (2009). An ultrastructural investigation of early Middle Pennsylvanian megaspores from the Illinois Basin, USA. Review of Palaeobotany and Palynology.

[ref-27] Gomez B, Martín–Closas C, Barale G, Thévenard F, Guignard G (2002). *Frenelopsis* (Coniferales: Cheirolepidiaceae) and related male organ genera from the Lower Cretaceous of Spain. Palaeontology.

[ref-28] Jung W (1967). Eine neue reconstruktion des Fruchtzapfens of Cheirolepis munsteri (Schenk). Neues Jahrbuch für Geologie und Paläontologie.

[ref-29] Jung W (1968). *Hirmerella munsteri* (Schenk) Jung N. Comb., Eine Bedeutsame Konifere Des Mesozikums. Palaeontographica.

[ref-30] Lupia R, Lidgard S, Crane PR (1999). Comparing palynological abundance and diversity: implications for biotic replacement during the Cretaceous angiosperm radiation. Paleobiology.

[ref-31] Miller CN (1999). Implications of fossil conifers for the phylogenetic relationships of living families. Botanical Review.

[ref-32] Needham JE (2011). Forest of the dinosaurs.

[ref-33] Philippe M, Bamford MK (2008). A key to morphogenera used for Mesozoic conifer-like woods. Review of Palaeobotany and Palynology.

[ref-34] Philippe M, Billon-Bruyat J-P, Garcia-Ramos JC, Bocat L, Gomez B, Piñuela L (2010). New occurrences of the wood *Protocupressinoxylon purbeckensis* Francis: implications for terrestrial biomes in South-western Europe at the Jurassic/Cretaceous boundary. Palaeontology.

[ref-35] Rees PM, Noto CR, Parrish MJ, Parrish JT (2004). Late Jurassic climates, vegetation, and dinosaur distributions. Journal of Geology.

[ref-36] Rees PM, Ziegler AM, Valdes PJ, Huber BT, MacLeod KG, Wing SL (2000). Jurassic phytogeography and climates: new data and model comparisons. Warm climates in earth history.

[ref-37] Sanchez S, Ahlberg PE, Trinajstic KM, Mirone A, Tafforeau P (2012). Three-dimensional synchrotron virtual paleohistology: a new insight into the world of fossil bone microstructures. Microscopy and Microanalysis.

[ref-38] Scott AC, Galtier J, Gostling NJ, Smith SY, Collinson ME, Stampanoni M, Marone F, Donoghue PCJ, Bengtson S (2009). Scanning electron microscopy and synchrotron radiation X-ray tomographic microscopy of 330 million year old charcoalified seed fern fertile organs. Microscopy And Microanalysis.

[ref-39] Sellwood BW, Valdes PJ (2008). Jurassic climates. Proceedings of the Geologists’ Association.

[ref-40] Slater BJ, McLoughlin S, Hilton J (2011). Guadalupian (Middle Permian) megaspores from a permineralized peat in the Bainmedart Coal Measures, Prince Charles Mountains, Antarctica. Review of Palaeobotany and Palynology.

[ref-41] Smith SY, Collinson ME, Rudall PJ, Simpson DA, Marone F, Stampanoni M (2009). Virtual taphonomy using synchrotron tomographic microscopy reveals cryptic features and internal structure of modern and fossil plants. Proceedings of the National Academy of Sciences of the United States of America.

[ref-42] Smith SY, Stockey RA (2001). A new species of Pityostrobus from the lower Cretaceous of California and its bearing on the evolution of Pinaceae. International Journal of Plant Sciences.

[ref-43] Smith SY, Stockey RA (2002). Permineralized pine cones from the Cretaceous of Vancouver Island, British Columbia. International Journal of Plant Sciences.

[ref-44] Spencer ART, Hilton J, Sutton MD (2013). Combined methodologies for three-dimensional reconstruction of fossil plants preserved in siderite nodules: *Stephanospermum braidwoodensis* nov. sp. (Medullosales) from the Mazon Creek lagerstätte. Review of Palaeobotany and Palynology.

[ref-45] Stockey RA (1977). Reproductive biology of the Cerro Cuadrado (Jurassic) fossil conifers: *Pararaucaria patagonica*. American Journal of Botany.

[ref-46] Stockey RA, Rothwell GW (2013). *Pararaucaria carrii* sp nov., anatomically preserved evidence for the conifer family Cheirolepidiaceae in the Northern Hemisphere. International Journal of Plant Sciences.

[ref-47] Strullu-Derrien C, Kenrick P, Tafforeau P, Badel E, Cochard H, Bonnemain J-L, Le Hérissé A, Lardeux H (2014). The earliest wood and its hydraulic properties documented in ca 407 million-year-old fossils using synchrotron microtomography. Botanical Journal of the Linnean Society.

[ref-48] Sutton MD, Garwood RJ, Siveter DJ, Siveter DJ (2012). SPIERS and VAXML: a software toolkit for tomographic visualisation and a format for virtual specimen interchange. Palaeontologia Electronica.

[ref-49] Sutton MD, Rahman IA, Garwood RJ (2014). Techniques for virtual palaeontology.

[ref-50] Taylor TN, Taylor EL, Krings M (2009). Paleobotany: the biology and evolution of fossil plants.

[ref-51] Watson J, Beck CB (1988). The Cheirolepidiaceae. Origin and evolution of gymnosperms.

[ref-52] West IM (1975). Evaporites and associated sediments of the basal Purbeck Formation (Upper Jurassic) of Dorset. Proceedings of the Geologists’ Association.

[ref-53] Wieland GR (1929). The world’s two greatest petrified forests. Science.

[ref-54] Wieland GR (1935). The Cerro Cuadrado petrified forest.

[ref-55] Wimbledon WA (1976). The Portland Beds (Upper Jurassic) of Wiltshire. Wiltshire Natural History Magazine.

[ref-56] Wimbledon WA, Cope JCW (1980). Portlandian correlation chart. A correlation of Jurassic rocks in the British Isles. Geological Society Special Report.

[ref-57] Wright JK, Cox BM (2001). British upper jurassic stratigraphy.

