# Supplementary material for: X-ray Synchrotron Microtomography of a silicified Jurassic Cheirolepidiaceae (Conifer) cone: histology and morphology of Pararaucaria collinsonae sp. nov
Source: PeerJ. 2014 Oct 23;2:e624. doi: 10.7717/peerj.624 (PMC4217189; doi:10.7717/peerj.624)
Supplement: Data S1 — Palaeogeographical Data based on literature survey. This file contains the results, dataset, and references from the literature survey. [file peerj-02-624-s001.pdf]

Supplemental Data S1

A literature survey was carried out to plot the palaeogeographic distribution of all published male and female cones for the Cheirolepidiaceae. Palaeogeographic latitude and longitudes were calculated using PointTracker after determining modern geographical co-ordinates from topographic and geological maps in tandem with Google Earth™. These were plotted using Corel Creative Suite X5™ on to a series of Mesozoic palaeomaps showing individual time slices. The palaeomaps where based on global palaeo-reconstruction maps by Ron Blakely (Blakey, 2008).

This file contains the results, dataset, and references from the literature survey.

Results

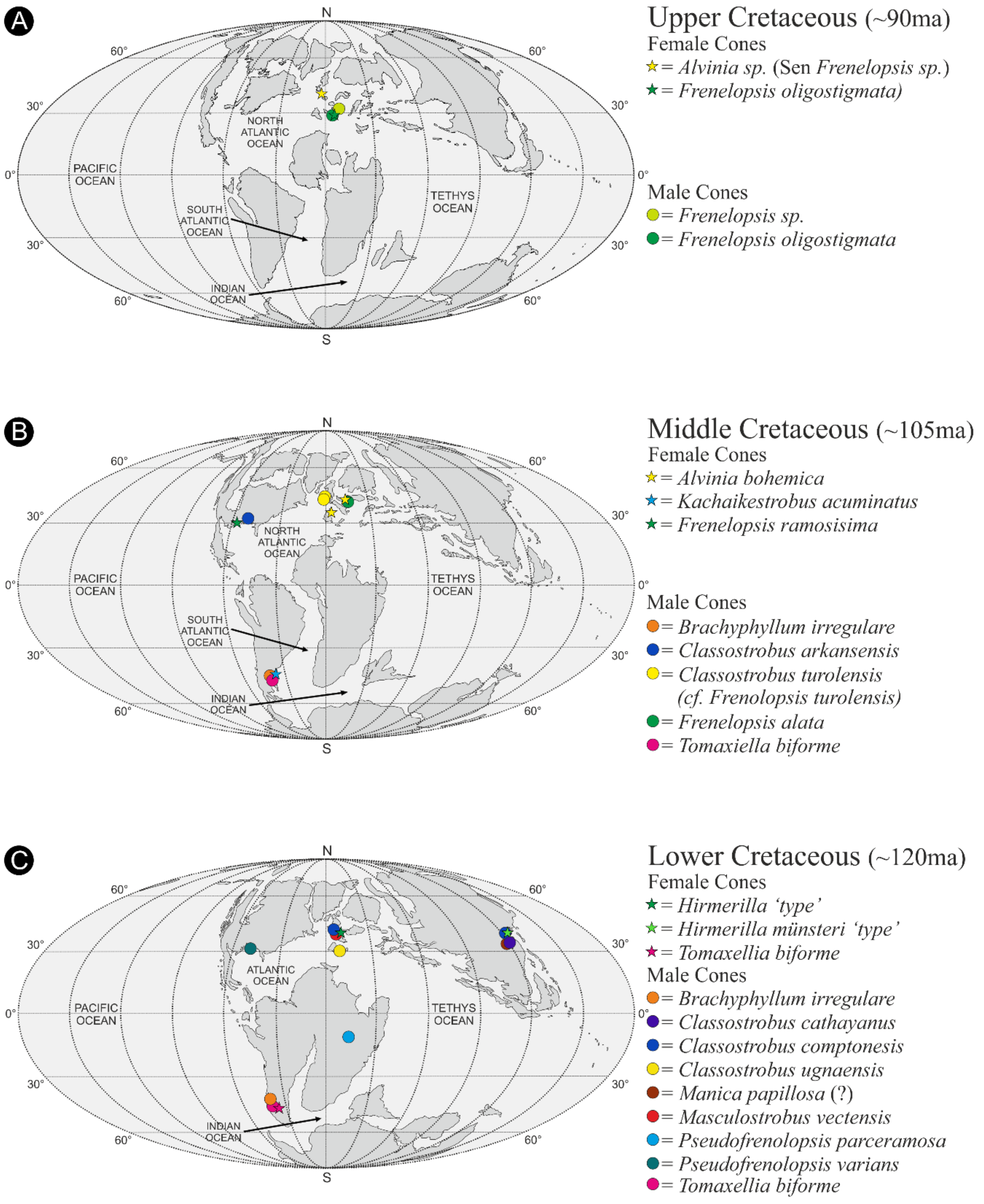
A
N
S
60°
30°
0°
NORTH ATLANTIC OCEAN
PACIFIC OCEAN
SOUTH ATLANTIC OCEAN
TETHYS OCEAN
INDIAN OCEAN
Upper Cretaceous (~90ma)
Female Cones
= *Alvinia sp.* (Sen *Frenelopsis sp.*)
= *Frenelopsis oligostigmata)*
Male Cones
= *Frenelopsis sp.*
= *Frenelopsis oligostigmata*
B
N
S
60°
30°
0°
NORTH ATLANTIC OCEAN
PACIFIC OCEAN
SOUTH ATLANTIC OCEAN
TETHYS OCEAN
INDIAN OCEAN
Middle Cretaceous (~105ma)
Female Cones
= *Alvinia bohemica*
= *Kachaikestrobus acuminatus*
= *Frenelopsis ramosisima*
Male Cones
= *Brachyphyllum irregulare*
= *Classostrobus arkansensis*
= *Classostrobus turolensis*
(cf. Frenolopsis turolensis)
= *Frenelopsis alata*
= *Tomaxiella biforme*
C
N
S
60°
30°
0°
ATLANTIC OCEAN
PACIFIC OCEAN
TETHYS OCEAN
INDIAN OCEAN
Lower Cretaceous (~120ma)
Female Cones
= *Hirmerilla 'type'*
= *Hirmerilla münsteri 'type'*
= *Tomaxellia biforme*
Male Cones
= *Brachyphyllum irregulare*
= *Classostrobus cathayanus*
= *Classostrobus comptonesis*
= *Classostrobus ugnaensis*
= *Manica papillosa* (?)
= *Masculostrobus vectensis*
= *Pseudofrenolopsis parceramosa*
= *Pseudofrenolopsis varians*
= *Tomaxellia biforme*

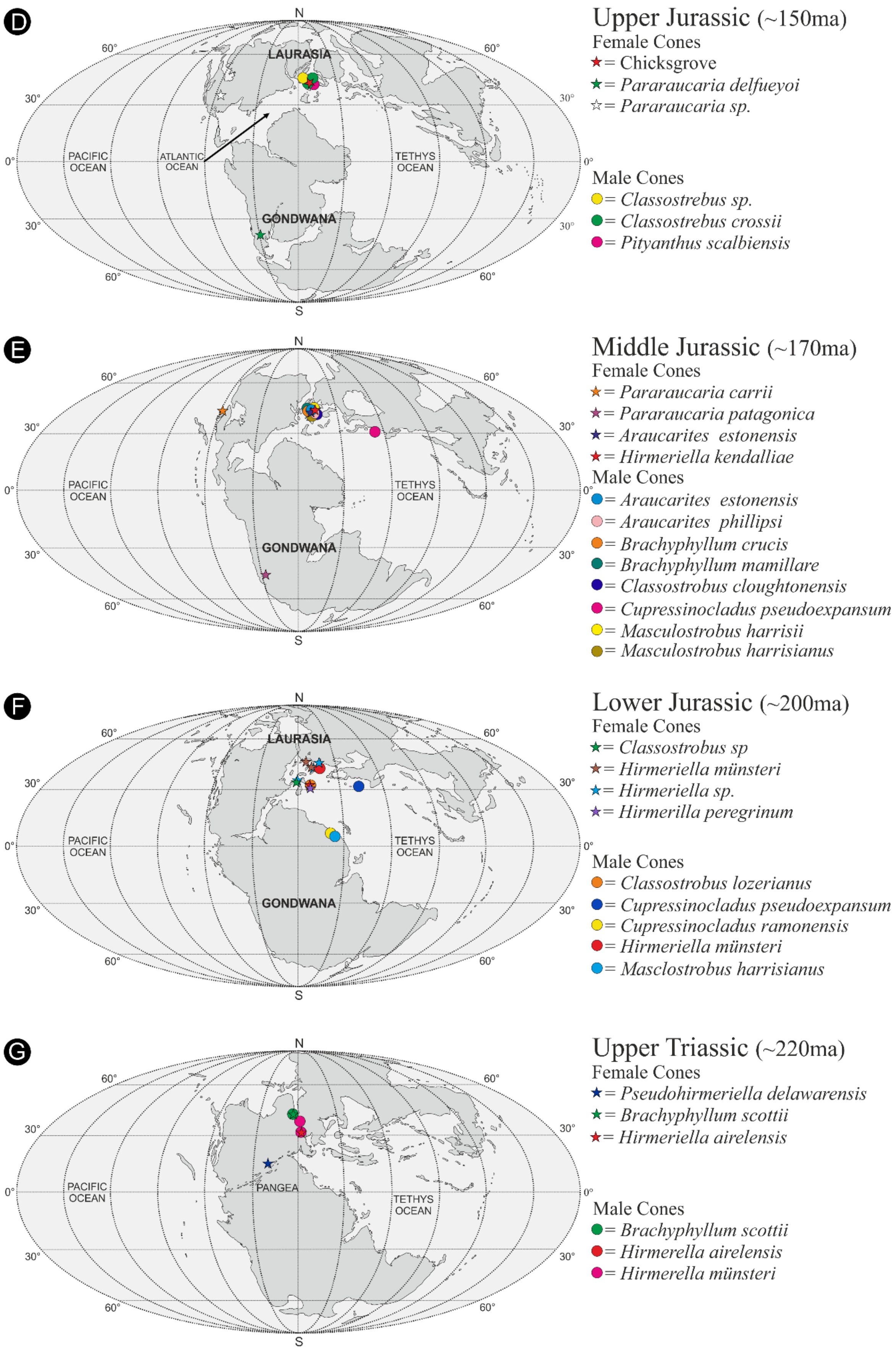
D
N
S
60°
30°
0°
LAURASIA
GONDWANA
PACIFIC OCEAN
ATLANTIC OCEAN
TETHYS OCEAN
Upper Jurassic (~150ma)
Female Cones
= Chicksgrove
= *Pararaucaria delfueyoi*
= *Pararaucaria sp.*
Male Cones
= *Classostrebus sp.*
= *Classostrebus crossii*
= *Pityanthus scalbiensis*
E
N
S
60°
30°
0°
GONDWANA
PACIFIC OCEAN
TETHYS OCEAN
Middle Jurassic (~170ma)
Female Cones
= *Pararaucaria carrii*
= *Pararaucaria patagonica*
= *Araucarites estonensis*
= *Hirmeriella kendalliae*
Male Cones
= *Araucarites estonensis*
= *Araucarites phillipsi*
= *Brachyphyllum crucis*
= *Brachyphyllum mamillare*
= *Classostrobus cloughtonensis*
= *Cupressinocladus pseudoexpansum*
= *Masculostrobus harrisii*
= *Masculostrobus harrisianus*
F
N
S
60°
30°
0°
LAURASIA
GONDWANA
PACIFIC OCEAN
TETHYS OCEAN
Lower Jurassic (~200ma)
Female Cones
= *Classostrobus sp*
= *Hirmeriella münsteri*
= *Hirmeriella sp.*
= *Hirmerilla peregrinum*
Male Cones
= *Classostrobus lozerianus*
= *Cupressinocladus pseudoexpansum*
= *Cupressinocladus ramonensis*
= *Hirmeriella münsteri*
= *Masclostrobus harrisianus*
G
N
S
60°
30°
0°
PANGEA
PACIFIC OCEAN
TETHYS OCEAN
Upper Triassic (~220ma)
Female Cones
= *Pseudohirmeriella delawarensis*
= *Brachyphyllum scottii*
= *Hirmeriella airelensis*
Male Cones
= *Brachyphyllum scottii*
= *Hirmerella airelensis*
= *Hirmerella münsteri*

Dataset

* APT = Age used in PointTracker

Paper	Genus/species	Location	Palaeo-location	Age	APT*	Organ
Alvin, Fraser & Watson, 1981	*Hirmeriella 'type'*	Hanover Point/Isle of Wright (UK) 50.65;-1.47	41.50; 08.66	Barremian, 129.4 to 125 MA	130 MA	♀ Cone
Alvin, Watson and Spicer, 1994	*Classastrobus comptonensis (rediscribed)*	Hanover Point/Isle of Wright (UK) 50.65;-1.47	41.50; 08.66	Barremian, 129.4 to 125 MA	130 MA	♂ Cone
Alvin, Spicer & Warson, 1978	*Pseudofrenolopsis parceramosa*	Compton bay/Hanover Point (UK) 50.65;-1.47	41.50; 08.66	Barremian, 129.4 to 125.0 MA	130 MA	♂ Cone
Alvin, Spicer & Watson, 1981	*Classostrobus comptonensis*	Hanover Point/Isle of Wright (UK) 50.65;-1.47	41.50; 08.66	Barremian, 129.4 to 125 MA	130 MA	♂ Cone
Alvin, Watson and Spicer, 1994	*Masculostrobus vectenis*	Hanover Point/Isle of Wright (UK) 50.65;-1.47	41.50; 08.66	Barremian, 129.4 to 125 MA	130 MA	♂ Cone
Archangelsky & Gamerra, 1967	*Tomaxiella biforme*	Argentina (Baquero Fm) -46.93; -69.31	-50.85; -39.23	Aptian, 125 to 113 MA (Cesari, et al. 2011 Radiometric - 114.67 MA)	110 MA	♂ Cone
Archangelsky & Gamerra, 1967	*Brachyphyllum irregulare*	Argentina (Baquero Fm) -46.93; -69.31	-50.85; -39.23	Aptian, 125 to 113 MA (Cesari, et al. 2011 Radiometric - 114.67 MA)	110 MA	♂ Cone
Archangelsky, 1966	*Tomaxiella biforme*	Bajo Grande Fm/Santa Cruz (Argentina) -46.93;-69.32	-47.45; -34.49	Aptian, 125 to 113 MA (Cesari, et al. 2011 Radiometric - 114.67 MA)	120 MA	♂ Cone
Archangelsky, 1968	*Tomaxiella biforme*	Bajo Grande Fm/Santa Cruz (Argentina) -46.93;-69.32	-47.45; -34.89	Aptian, 125 to 113 MA (Cesari, et al. 2011 Radiometric - 114.67 MA)	120 MA	♂ Cone
Archangesky, 1963	*Tomaxiella biforme*	Bajo Grande Fm/Santa Cruz (Argentina) -46.93;-69.32	-47.45; -34.89	Aptian, 125 to 113 MA (Cesari, et al. 2011 Radiometric - 114.67 MA)	120 MA	♂ Cone
Archangesky, 1963	*Brachyphyllum irregulare*	Bajo Grande Fm/Santa Cruz (Argentina) -46.93;-69.32	-47.45; -34.89	Aptian, 125 to 113 MA (Cesari, et al. 2011 Radiometric - 114.67 MA)	120 MA	♂ Cone
Axesmith & Jacobs, 2005	*Frenelopsis ramosissima*	Jons Ranch/Texas (USA) 32.25; -97.81	30.83; -56.35	Aptian/Albian boundary, ~113 MA	110 MA	♂ and ♀ Cones
Axesmith, Kriggs & Wesselkov, 2004	*Classostrobus arkansensis* (Sen *P. paraceramosa*)	Howard County/Nasville (USA) 34.07; -93.84	31.72; -51.02	Aptian/Albian boundary, ~113 MA	110 MA	♂ Cone
Axsmith, Andrews & Fraser, 2004	*Pseudohirmerella delawarensis*	Smith Clark Quarry/Milford NJ (USA) 40.57;-75.14	14.86; -20.01	Norian, 227 to 208.5	220 MA	♀ Cone
Barale, 1987	*Classostrobus sp.*	St. Fromond (France) 49.22; -1.09	35.67; 02.99	Rhaetian/Hettangian 208.5 to 199.3 MA	200 MA	♂ and ♀ Cones
Barale, 1987	*Hirmeriella sp.*	St. Fromond (France) 49.22; -1.09	35.67; 02.99	Rhaetian/Hettangian 208.5 to 199.3 MA	200 MA	♂ and ♀ Cones
Barale, Marron & Ramis, 1976	*Frenelopsis sp.*	Torrelaguna/Madrid (Spain) 40.84; -3.53	30.94; -00.72	Senonian 89.3 to 65.5 MA	80 MA	♂ Cone
Barnard & Miller, 1975	*Cupressinocladus psuedoexpansum*	North Iran 38.8; 46.9	29.15; 54.79	Bajocian (Shem Shak Fm) 170.3 to 168.3 MA	170 MA	♂ Cone

Paper (cont…)	Genus/species (cont…)	Location (cont…)	Palaeo-location (cont…)	Age (cont…)	APT* (cont…)	Organ (cont…)
Carpentier, 1937	*Frenelopsis sp.*	Saint Baume Massif (France) 43.32; 5.76	33.12; 07.63	Lower Campanian, 83.6 to 72.1 MA	80 MA	♂ Cone
Chaloner & Lorch, 1960	*Cupressinocladus ramonensis*	Israel 30.57;34.91	11.58; 27.08	Rhaetian/Liassic, 208.5 to 199.3 MA	200 MA	♂ Cone
Lorch, 1968	*Cupressinocladus ramonensis*	Israel 30.57;34.91	11.58; 27.08	Rhaetian/Liassic, 208.5 to 199.3 MA	200 MA	♂ Cone
Cleal, 2003	*Classostrobus sp.*	Stonesfield slate (UK) 51.85; -1.43	40.80; 09.50	Bathonian, 168.3 to 166.1 MA	170 MA	♂ Cone
Clement-Westerhoff & Van Konijnenburg-Van Cittert, 1991	*Hirmeriella muensteri*	Unternschreez - Bayreuth (Germany) 49.95; 11.57	39.85; 12.85	Hettangian, 201.3 to 199.3 MA	200 MA	♂ Cone
Daviero, et al., 2001	*Frenelopsis turolensis*	Rubielos de Mara (Spain) 40.19; -0.65	28.76; 05.94	Middle Albian, 113 to 100.5 MA	110 MA	♂ Cone (immature cone)
Del Fueyo, et al., 2008	*Kachaikestrobus acuminatus*	Santa Cruz Provence (Argentina) ~-42.9;-67.08	-46.83; -37.18	Albian, 113 to 100.5 MA	110 MA	♀ Cone
Escapa, et al., 2013	*Pararacaria delfueyoi*	Chubut Prvence (Argentina) -43.19;-69.22	-39.79; -29.71	Oxfordian/Kimmeridgian, 157.5 MA (Radiometric)	160 MA	♀ Cone
Fontaine, 1893; Watson, 1977	*Pseodofrenelopsis varians*	Glen Rose/Texas/USA 32.23, -97.76	30.1; -50.36	Aptian/Lower most Albian 125 - ~113 MA	120 MA	♂ Cone
Francis, 1983	*Classostrobus sp.*	Purbeck/Dorset (UK) 50.53;-2.44	44.14; 01.44	Tithonian, 152.1 to 145 MA	150 MA	♂ Cone
Gee, et al, 2014	*Pararaucaria sp.*	Morrison Formation (USA) 38.82; -110.41	35.63,-58.43	Kimmeridian to Tithonian, 157 to 145 Ma	150 MA	♀ Cone
Gomez, et al., 2002	*Classostrobus ugnaensis* (c.f. *F. ugnaenisis*)	Cuenca (Spain) 40.01; -2.14	30.88; 07.85	Upper Barremian 129.4 to 125 MA	130 MA	♂ Cone
Gomez, et al., 2002	*Classostrobus turolensis* (c.f. *F. turolensis*)	Teruel (Spain) 40.35; -1.11	28.76; 05.94	Lower/Middle Albian, 113 to 100.5 MA	110 MA	♂ Cone
Harris, 1957	*Cheirolepis munsteri*	Glamorgan (Wales, UK) 51.49; -3.26	38.03; 00.18	Rhaetian, 208.5 to 201.3 MA	200 MA	♂ Cone
Harris, 1979	*Brachyphyllum crucis*	Yorkshire(UK) 53.7; -1.56	42.72; 08.40	Bajocian to Bathonian, 170.3 to 166.1 MA	170 MA	♂ Cone
Hirmer & Horhamer, 1934	*Hirmeriella muensteri*	Germany/Wales 10; 16	39.58; 11.60	Rhaetian/Liassic, 208.5 to 199.3 MA	200 MA	♂ and ♀ Cones
HlustiK & K0nzalova, 1976	*Frenelopsis alata*	Hlochetin (Prague-Czech Republic) 51.11;14.54	40.17; 14.13	Cenomanian, 100.5 to 93.9 MA	100 MA	♂ Cone
Horhamer, 1933 (see also Jung, 1967)	*Hirmeriella muensteri*	Germany/Wales 10; 16	39.58; 11.60	Rhaetian/Liassic, 208.5 to 199.3 MA	200 MA	♂ and ♀ Cones
Jung, 1967	*Cheirolepis munsteri* (name changed to *Hirmerella munsteri*)	Schnaittach/Nurmberg region Germany 49.5; 11.3	39.58; 11.60	Rhaetian/Liassic, 208.5 to 199.3 MA	200 MA	♀ (seedless) Cones
Jung, 1967	*Hirmerella*	Altdorf, Germany 49.39, 11.36	38.67; 12.12	Rhaetian–Toarcian 208.5-174.1	200 MA	Sterile Cone
Jung, 1968	*Hirmerella munsteri* (name changed from *Cheirolepis munsteri*)	Grossbellhofen,Franconia (Germany) 49.57; 11.31	39.58; 11.60	Rhaetian/Liassic, 208.5 to 199.3 MA	200 MA	♀ Cone

Paper (cont…)	Genus/species (cont…)	Location (cont…)	Palaeo-location (cont…)	Age (cont…)	APT* (cont…)	Organ (cont…)
Kendall, 1949	*Brachyphyllum scottii*	Larachberg/Morvern (Scotland) 56.57;-5.78	42.08; -04.27	Rhaetian/Liassic, 208.5 to 199.3 MA	210 MA	♂ and ♀ Cone, Cone scales and cone axies
Kendall, 1952	*Pagiophyllum connivens* (c.f. *Araucarites estonensis)*	Eston Moor/Yorkshire (UK) 54.55; -1.12	42.72; 08.40	Bajocian, 170.3 to 168.3 MA	170 MA	♂ and ♀ Cone
Kendall, 1952	*Araucarites phillipsi*	Whitby/Yorkshire (UK) 54.48; -0.61	42.72; 08.40	Bajocian, 170.3 to 168.3 MA	170 MA	♂ Cone
Kim, et al., 2012	*Classostrobus comptonensis*	Youngdong Group(South Korea) 36.20, 127.78	40.45; 127.07	Berriasian–Albian 145.0–100.5	130 MA	♂ Cone
Kim, et al., 2012	Unnamed (*Hirmerella münsteri* type)	Youngdong Group(South Korea) 36.20, 127.78	40.45; 127.07	Berriasian–Albian 145.0–100.5	130 MA	♀ Cone
Krassilov, 1982	*Hirmerella sp.*	Odrovaz (Poland) 49.49; 19.85	42.08; 18.49	Hettangian, 201.3 to 199.3 MA	200 MA	♀ Samara from Cone
Kvacek, 2000	*Frenelopsis alata* (♀ cones reassigned to *Alvinia bohemica*)	Pecinov Quarry (Czech Republic) 50.14;13.89	39.11; 13.45	Cenomanian(Middle), 100.5 to 93.9 MA	100 MA	♂ Cone
Kvacek, 2000	*Alvinia bohemica* (this is the reassigned name for ♀ cones of *Frenelopsis alata*)	Lipenêc(Czech Republic) 50.31, 13.71	39.11; 13.45	Cenomainian 100.5–93.9	100 MA	♀ Cone
Kvacek, 2000	*Alvinia bohemica*	Lipenêc(Czech Republic) 50.31, 13.71	39.11; 13.45	Cenomainian 100.5–93.9	100 MA	♀ Cone
Lorch, 1986	*Masclostrobus harrisianus*	Makhtesh Ramon(Israel) 30.61, 34.88	11.58; 27.08	Rhaetian–Pliensbachian 208.5–185.0	200 MA	♂ Cone
Muir & Van Konijnenburg-Van Cittert, 1970	*Hirmeriella airelensis*	Airel (France) 49.22; -1.08	32.92; 01.66	Norian to Hettangian 227 to 201 MA	210 MA	♂ and ♀ Cone scales and pollen
Pons & Broutin, 1978	*Frenelopsis oligostomata*	Portugal 38.69; -9.35	29.72; -05.53	Senonian 89.3 to 65.5 MA	80 MA	♂ and ♀ Cones
Pons, 1979	*Frenelopsis alata*	L'Anjou (France) 47.67; -0.21	36.44; 02.06	Cenomanian, 100.5 to 93.9 MA	100 MA	♂ and ♀ Cones
Rothwell, et al., 2007	*Classostrebus crossii*	Cirencester (UK) 51.77;-1.97	42.58; 08.02	Callovian, 166.1 to 163.5 MA	160 MA	♂ Cone
Rothwell, *et al.,* 2007	*Classostrobus crossii*	Freeth's Wood Gravel Pit/Ashton Keynes(UK) 51.64, -1.95	42.58; 08.01	Callovian 166.1–163.5	160 MA	♂ Cone
Stockey & Rothwell, 2013	*Pararacaria carrii*	Izee/Oregon (USA) 44.07;-119.37	37.80; -63.14	Callovian(Officer member), 166.1 to 163.5 MA	160 MA	♀ Cone
Stockey, 1977; Calder, 1953; Escapa, et al., 2012	*Pararacaria patagonia*	Cero Cuadrado (Argentina) -51.26;-72.33	-48.04; -29.91	La Matilde Fm; Callovian, 166.1 to 163.5	160 MA	♀ Cone
Thevenard, 1993	*Classostrobus lozerianus*	Chaldecoste outcrop/Lozere(France) 44.53, 3.49	32.89; 07.79	Hettangian–Sinemurian 201.3–190.8	200 MA	♂ Cone
Thevenard, 1993	*Hirmeriella airelensis*	Chaldecoste outcrop/Lozere(France) 44.53, 3.49	32.89; 07.79	Hettangian–Sinemurian 201.3–190.8	200 MA	♀ Cone Scale
Thevenard,1993	*Himeriella peregrinum*	Chaldecoste outcrop/Lozere(France) 44.53, 3.49	32.89; 07.79	Hettangian–Sinemurian 201.3–190.8	200 MA	♀ Cone Scale

Paper (cont…)	Genus/species (cont…)	Location (cont…)	Palaeo-location (cont…)	Age (cont…)	APT* (cont…)	Organ (cont…)
Van Cittert, 1971	*Masculostrobus harrisii*	Yorkshire (UK) 53.7; -1.56	42.72; 08.40	Bajocian to Bathonian, 170.3 to 166.1 MA	170 MA	♂ Cone
Van Cittert, 1971	*Brachyphyllum crucis*	Yorkshire (UK) 53.7; -1.56	42.72; 08.40	Bajocian to Bathonian, 170.3 to 166.1 MA	170 MA	♂ Cone
Van Konijnenburg-Van Cittert, 1971	*Brachyphyllum crucis*	Lower Deltaic/Hasty Bank/Yorkshire(UK) 54.42, -1.13	42.78; 09.19	Aalenian 174.1–170.3	170 MA	♂ Cone
Van Konijnenburg-Van Cittert, 1971	*Pagiophyllum connivens*, (c.f. *Pagiophyllum kurri*)	Lower/Middle Deltaic/Eston Moor/Yorkshire(UK) 54.55, -1.12	43.78; 09.03	Bajocian 170.3–168.3	170 MA	♂ Cone
Van Konijnenburg-Van Cittert, 1971	*Masclostrobus harrisianus*	Middle Deltaic/Cayton Bay/Yorkshire(UK) 54.24, -0.36	42.86 09.99	Aalenian–Bajocian 174.1–168.3	170 MA	♂ Cone
Van Konijnenburg-Van Cittert, 1971	*Brachyphyllum mamillare*, (pos. Araucaria)	Lower-Middle-Upper Deltaic/Whitby/Yorkshire(UK) 54.49, -0.63	42.78; 09.19	Aalenian–Bathonian 174.1–166.1	170 MA	♂ Cone
Van Konijnenburg-Van Cittert, 1987	*Classostrobus cloughtonensis*	Cloughton Wykes, Yorkshire(England) 54.34, -0.45	42.86; 09.19	Aalenian–Bajocian 174.1–168.3	170 MA	♂ Cone
Van Konijnenburg-Van Cittert, 1987	*Hirmeriella kendalliae*	Cloughton Wykes, Yorkshire(England) 54.34, -0.45	42.86; 09.19	Aalenian–Bajocian 174.1–168.3	170 MA	♀ Cone
Van Konijnenburg-Van Cittert,1971	*Pityanthus scalbiensis* (family assignation dubious)	Upper Deltaic/Scalby Wake/Yorkshire(UK) 54.32, -0.45	44.64; 09.51	Bathonian 168.3–166.1	160 MA	♂ Cone
Wason & Alvin, 1976	*Pseudofrenelopsis paraceramosa*	Jebel Dirra/Eastern Daefur(Sudan) 13.59; 26.06	-05.36; 6.44	Berriasian to Albian (estimate in Watson 1982), 145-100.5	130 MA	♂ or ♀ Cones not specified (check again)
Zhou, 1983	*Classostrobus cathayanus*	Qiya Town/Nanjing/China 32.00, 118.60	35.52; 120.5	Lower Cretaceous ~113-145 MA	120MA	♂ Cone
Zhou, 1995	*Manica papillosa*	Suqing/Xinchang/Zhejiang/China 29.40,120.9	34.18; 122.6	Lower Cretaceous ~113-145 MA	120MA	♂ Cone

References

Alvin KL, Fraser CJ, and Spicer RA. 1981. Anatomy and paleoecology of *Pseudofrenelopsis* and associated conifers in the English Wealden. *Palaeontology* 24:759-778.

Alvin KL, Spicer RA, and Watson J. 1978. A *Classopollis*-containing male cone associated with *pseudofrenelopsis*. *Palaeontology* 21:847-856.

Alvin KL, Watson J, and Spicer RA. 1994. A new coniferous male cone from the english wealden and a discussion of pollination in the cheirolepidiaecae. *Palaeontology* 37:173-180.

Archangelsky S. 1963. A new Mesozoic flora from Tico, Santa Cruz Provence Argentina. *Bulletin of the British Museum of Natural History* 8:48-92.

Archangelsky S. 1966. New Gymnosperms from the Tico Flora, Santa Cruz Provence, Argentina. *Bulletin of the British Museum of Natural History* 13:259-295.

Archangelsky S. 1968. On the genus *Tomaxellia* (Coniferae) from the Lower Cretaceous of Patagonia (Argentina) and its male and female cones. *Botanical Journal of the Linnean Society* 61:153-165.

Archangelsky S, and Gamerro JC. 1967. Pollen grains found in coniferous cones from the Lower Cretaceous of Patagonia. *Review of Palaeobotany and Palynology* 5:179-182.

Axsmith BJ, Andrews FM, and Fraser NC. 2004. The structure and phylogenetic significance of the conifer Pseudohirmerella delawarensis nov comb. from the Upper Triassic of North America. *Review of Palaeobotany and Palynology* 129:251-263.

Axsmith BJ, and Jacobs BF. 2005. The conifer Frenelopsis ramosissima (Cheirolepidiaceae) in the Lower Cretaceous of Texas: Systematic, biogeographical, and paleoecological implications. *International Journal of Plant Sciences* 166:327-337.

Axsmith BJ, Krings M, and Waselkov K. 2004. Conifer pollen cones from the Cretaceous of Arkansas: Implications for diversity and reproduction in the Cheirolepidiaceae. *Journal of Paleontology* 78:402-409.

Barale G. 1987. Cheirolepidiaceae from the lower jurassic of saint-fromond, carentan basin (manche, france). *Bulletin De La Societe Botanique De France-Actualites Botaniques* 134:19-37.

Barale G, Marron TF, and Ramis CA. 1988. Study of male cones of frenelopsis schenk emend reymanowna and watson 1976 (cheirolepidiaceae) in upper cretaceous from torrelaguna (madrid province spain). *Geobios* 21:187-199.

Barnard PDW. 1968. A new species of *Masculostrobus* Seward producing *Classopollis* pollen from the Jurassic of Iran. *Botanical Journal of the Linnean Society* 61:167-176.

Barnard PDW, and Miller JC. 1976. Flora of the Shemshak formation (Elburtz, Iran), Part 3: Middle Jurassic (Dogger) plants from Katumbargah, Vasek Gah and Imam Manak. *Palaeontographica Abteilung B-Palaophytologie* 155:31-117.

Calder MG. 1953. A Coniferous petrified forest in Patagonia. *Bulletin of the British Museum of Natural History* 2:99-138.

Carpentier AM. 1937. Empreintes do *Frenelopsis* trouvees dans le Campanien Inferieur du Massif de la Sainte-Baume. *Annales du Musee D'Histoire Naturelle de Marseile* 28:4-15.

Chaloner WG, and Lorch J. 1960. An opposite-leafed conifer from the Jurasic of Israel. *Palaeontology* 2:236-242.

Cleal CJ, and Rees PM. 2003. The Middle Jurassic flora from Stonesfield, Oxfordshire, UK. *Palaeontology* 46:739-801.

Clement-Westerhof JA, and Van Konijnenburg Van Cittert JHA. 1991. Hirmeriella-muensteri - new data on the fertile organs leading to a revised concept of the cheirolepidiaeae. *Review of Palaeobotany and Palynology* 68:147-179.

Daviero V, Gomez B, and Philippe M. 2001. Uncommon branching pattern within conifers: Frenelopsis turolensis, a Spanish Early Cretaceous Cheirolepidiaceae. *Canadian Journal of Botany-Revue Canadienne De Botanique* 79:1400-1408.

Del Fueyo GM, Archangelsky S, Llorens M, and Cuneo R. 2008. Coniferous ovulate cones from the Lower Cretaceous of Santa Cruz Province, Argentina. *International Journal of Plant Sciences* 169:799-813.

Escapa IH, Cuneo NR, Rothwell G, and Stockey RA. 2013. *Pararaucaria delfueyoi* sp nov from the Late Jurassic Canadon Calcareo Formation, Chubut, Argentina: Insights into the evolution of the Cheirolepidiaceae. *International Journal of Plant Sciences* 174:458-470.

Escapa IH, Rothwell GW, Stockey RA, and Cuneo NR. 2012. Seed cone anatomy of Cheirolepidiaceae (coniferales): Reinterpreting *Pararaucaria patagonica* wieland. *American Journal of Botany* 99:1058-1068.

Fontaine WM. 1893. Notes on some fossil plants from the Trinity Division of the Comanche Series of Texas. *Proceedings of the United States National Museum* 16:261-282.

Francis JE. 1983. The dominant conifer of the Jurassic Purbeck Formation, England. *Palaeontology* 26:277-294.

Gee CT, Dayvault RD, Stockey RA, and Tidwell WD. 2014. Greater palaeobiodiversity in conifer seed cones in the Upper Jurassic Morrison Formation of Utah, USA. *Palaeobiodiversity and Palaeoenvironments* 94:363–375.

Gomez B, Martin-Closas C, Barale G, De Porta NS, Thevenard F, and Guignard G. 2002. Frenelopsis (Coniferales : Cheirolepidiaceae) and related male organ genera from the Lower Cretaceous of Spain. *Palaeontology* 45:997-1036.

Harris TM. 1957. A Liassico-Rhaetic flora in South Wales. *Proceedings of the Royal Society of London Series B* 147:289-308.

Harris TM. 1979. *The Yorkshire Jurassic Flora, Volume 5, Coniferales*. London: British Museum (Natural History).

Hirmer M, and Horhamer L. 1934. Zur weiterem Kenntnis von *Cheirolepis* Schimper und *Hirmerella* Horhammer mit Bemerkungen uber deren systematische Konifere des Mesozoikums. *Palaeontographica Abteilung B-Palaophytologie* 79:67-84.

Hlustik A, and Konzalova M. 1976. Polliniferous cones of *Frenelopsis alata* (K. Feistm.) Knobloch from the Cenomanian of Czechoslovakia. *Vestnik Ustredniho ustavu geologickeho* 51:37-46.

Horhammer L. 1933. Über die Coniferen-Gattungen Cheirolepis Schimper und Hirmeriella nov. gen. aus dem Räth-Lias von Franken. *Bibliotheca Botanica* 107:1-33.

Jung W. 1967. Eine neue reconstruktion des Fruchtzapfens of *Cheirolepis munsteri* (Schenk). *Neues Jahrbuch für Geologie und Paläontologie* 2.

Jung W. 1968. *Hirmerella munsteri* (Schenk) Jung N. Comb., Eine bedeutsame konifere des Mesozikums. *Palaeontographica Abteilung B-Palaophytologie* 122:55-93.

Kendall MW. 1949. On a new conifer from the Scotish Lias. *Annals and Magazine of Natural History* 12:299-307.

Kendall MW. 1952. Some conifers from the Jurassic of England. *Annals and Magazine of Natural History* 12:583-594.

Kim J, Seo J, and Nam G. 2012. Pseudofrenelopsis cf. parceramosa and its reproductive

organs from the Lower Cretaceous Youngdong Group of Korea. *Geosciences Journal* 16:399-407.
Krassilov VA. 1982. On the ovuliferous organ of *Hirmerella*. In: Nautiyal DD, and Pant DD, editors. Phyta, Studies on Living and Fossil Plants, Pant Commemerative Volume. Allahabad (India): University of Allahabad. p 296.
Kvacek J. 2000. Frenelopsis alata and its microsporangiate and ovuliferous reproductive structures from the Cenomanian of Bohemia (Czech Republic, Central Europe). *Review of Palaeobotany and Palynology* 112:51-78.
Lorch J. 1968. Some Jurassic conifers from Israel. *Botanical Journal of the Linnean Society* 61:177-188.
Muir MD, and Van Konijnenburg-Van Cittert JHA. 1970. A Rhaeto-Liassic flora from Airel, northern France. *Palaeontology* 13:433-442.
Pons D. 1979. Les organenes reproducteurs de *Frenelopsis alata* (K. Feistm.) Knobloch, Cheirolepidiaceae du Cenomanien de L'Anjou, France. *CR 104e Congre`s National des Sociétés Savantes Science* 1:209-231.
Pons D, and Broutin J. 1978. Les organes reproducteurs de *Frenelopsis oligostomata* (Cretac, Portugal). *CR 103e Congre`s National des Sociétés Savantes Science* 2:139-159.
Rothwell GW, Mapes G, Hilton J, and Hollingworth NT. 2007. Pollen cone anatomy of Classostrobus crossii sp nov (Cheirolepidiaceae). *International Journal of Coal geology* 69:55-67.
Stockey RA, and Rothwell GW. 2013. *Pararaucaria carrii* sp nov., anatomically preserved evidence for the conifer family Cheirolepidiaceae in the Northern Hemisphere. *International Journal of Plant Sciences* 174:445-457.
Thevenard F. 1993. The Lower Jurassic Conifers of the Chaldecoste Outcrop, Causses Basin (Lozere, France). *Review of Palaeobotany and Palynology* 78:145-166.
Van Konijnenburg Van Cittert JHA. 1971. In situ gymnosperm pollen from the Middle Jurassic of Yorkshire. *Acta Botanica Neerlandica* 20:1-96.
Van Konijnenburg Van Cittert JHA. 1987. New data on *Pagiophyllum maculosum* Kendal and its male cone from the Jurassic of North Yorkshire. *Review of Palaeobotany and Palynology* 51:95-105.
Watson J. 1977. Some Lower Cretaceous conifers of the Cheirolepidiaceae from the U.S.A and England. *Palaeontology* 20:715-749.
Watson J, and Alvin KL. 1976. Silicone rubber casts of silicified plants from the Cretaceous of Sudan. *Palaeontology* 19:641-650.
Zhou Z. 1983. A heterophyllous cheirolepidiaceous conifer from the Cretaceous of East China. *Palaeontology* 26:789-811.
Zhou ZY. 1995. On some cretaceous pseudofrenelopsids with a brief review of cheirolepidiaceous conifers in china. *Review of Palaeobotany and Palynology* 84:419-438.
